# Nature of NMR Shifts in Paramagnetic Octahedral Ru(III) Complexes with Axial Pyridine-Based Ligands

**DOI:** 10.1021/acs.inorgchem.2c03282

**Published:** 2023-02-10

**Authors:** Jan Chyba, Anna Hruzíková, Michal Knor, Petra Pikulová, Kateřina Marková, Jan Novotný, Radek Marek

**Affiliations:** † CEITEC—Central European Institute of Technology, Masaryk University, Kamenice 5, CZ-62500 Brno, Czechia; ‡ Department of Chemistry, Faculty of Science, Masaryk University, Kamenice 5, CZ-62500 Brno, Czechia; § National Center for Biomolecular Research, Faculty of Science, Masaryk University, Kamenice 5, CZ-62500 Brno, Czechia; ∥ Institute of Inorganic Chemistry, Slovak Academy of Science, Dúbravská cesta 9, SK-84536 Bratislava, Slovakia

## Abstract

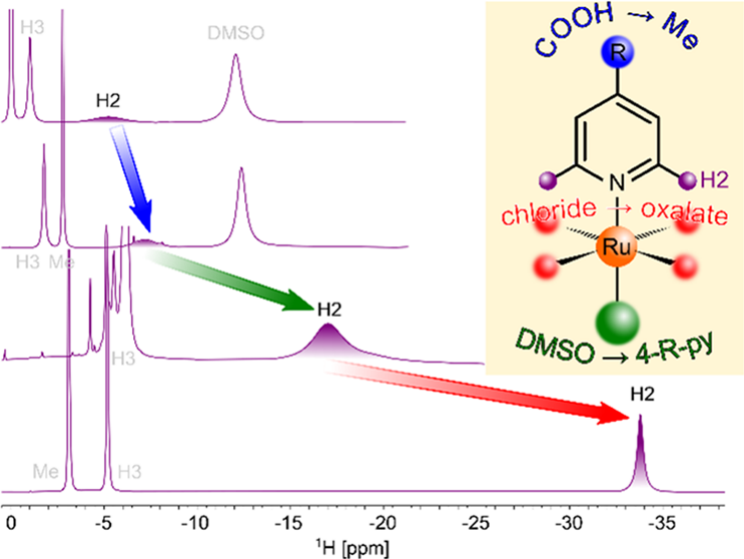

In recent decades, transition-metal coordination compounds have been extensively studied for their antitumor and antimetastatic activities. In this work, we synthesized a set of symmetric and asymmetric Ru(III) and Rh(III) coordination compounds of the general structure (Na^+^/K^+^/PPh_4_^+^/LH^+^) [*trans*-M^III^L(eq)*_n_*L(ax)_2_]^−^ (M = Ru^III^ or Rh^III^; L(eq) = Cl, *n* = 4; L(eq) = ox, *n* = 2; L(ax) = 4-R-pyridine, R = CH_3_, H, C_6_H_5_, COOH, CF_3_, CN; L(ax) = DMSO-*S*) and systematically investigated their structure, stability, and NMR properties. ^1^H and ^13^C NMR spectra measured at various temperatures were used to break down the total NMR shifts into the orbital (temperature-independent) and hyperfine (temperature-dependent) contributions. The hyperfine NMR shifts for paramagnetic Ru(III) compounds were analyzed in detail using relativistic density functional theory (DFT). The effects of (i) the 4-R substituent of pyridine, (ii) the axial *trans* ligand L(ax), and (iii) the equatorial ligands L(eq) on the distribution of spin density reflected in the “through-bond” (contact) and the “through-space” (pseudocontact) contributions to the hyperfine NMR shifts of the individual atoms of the pyridine ligands are rationalized. Further, we demonstrate the large effects of the solvent on the hyperfine NMR shifts and discuss our observations in the general context of the paramagnetic NMR spectroscopy of transition-metal complexes.

## Abstract 2

A series of octahedral ruthenium(III) compounds were synthesized and characterized using paramagnetic NMR spectroscopy. The effects of the para substituent of the pyridine ligand, the axial trans ligand, and the equatorial ligands at the ruthenium center, and the solvent on the hyperfine NMR shifts were observed and analyzed. The experimental NMR data were interpreted using relativistic DFT calculations.

## 1 Introduction

Octahedral Ru(III) coordination compounds have been shown to exhibit significant antitumor activities^1−3^ with large potential for regulating the migration of cells to treat the formation of metastases.^4^ There are two extensively investigated classes of octahedral Ru(III) complexes—asymmetric Q^+^[*trans*-Ru^III^Cl_4_L(DMSO-*S*)]^−^ and symmetric Q^+^[*trans*-Ru^III^Cl_4_L_2_]^−^. The exact mode of action of these compounds is not known, but it is assumed that they are (bio)activated by the hydrolysis of equatorial chloride(s)^1^ and can, therefore, be classified as stimuli-responsive metallodrugs (prodrugs).^5^

Ruthenium(III) compounds are paramagnetic in nature as they bear an unpaired electron at the metal center. Therefore, their characterization by nuclear magnetic resonance (NMR) spectroscopy is nontrivial. Theoretical calculations of the NMR shifts of the ligands are frequently required to predict and interpret experimental observations and to help with assigning the ^1^H and ^13^C NMR resonances in these paramagnetic compounds.^6−9^ In the last two decades, there has been significant progress in the theoretical prediction of the NMR shifts of paramagnetic systems.^10−14^ The methodology is particularly well developed for electronic doublets, including Ru(III) compounds.^15,16^ However, the calculation of NMR shifts for paramagnetic transition-metal complexes still represents a frontier in modern quantum chemistry.

**Figure 1:**
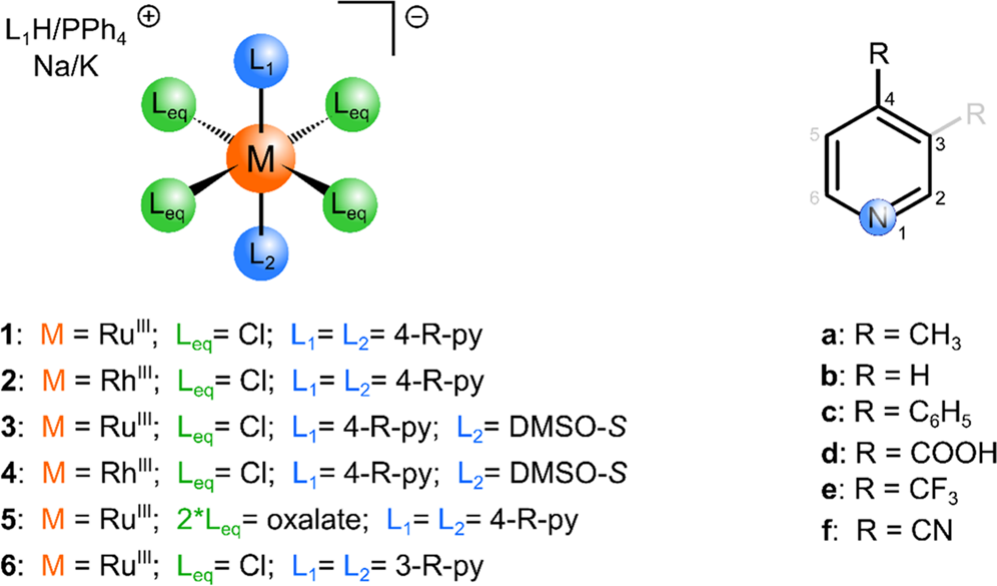
Structure and atom numbering scheme of ruthenium (**1**, **3**, **5**, **6**) and rhodium (**2**, **4**) coordination compounds with the octahedral complex anion [*trans*-MCl_4_L_2_]^−^ (**1**, **2**, **6**), [*trans*-MCl_4_(DMSO-*S*)L]^−^ (**3**, **4**), or [*trans*-Ru(ox)_2_L_2_]^−^ (**5**), containing pyridine-based ligands (**a**–**f**) in axial positions and the specified counter-cation.

In this account, we describe the synthesis and characterization of symmetric octahedral Ru(III) complexes (Figure 1) with two identical axial pyridine-based ligands and investigate the coordination exchange of ligands with water in an aqueous environment. Symmetric Q^+^[*trans*-Ru^III^Cl_4_L_2_]^−^ compounds are compared with their diamagnetic Rh(III) analogs and with the previously reported asymmetric Q^+^[*trans*-Ru^III^Cl_4_L(DMSO-*S*)]^−^ systems.^8^ Experimental paramagnetic NMR spectroscopy is used to characterize newly synthesized compounds. The effects of the R substituent in a 4-R-pyridine axial ligand, the equatorial ligands, and the type of solvent on the distribution of the spin density reflected in the hyperfine contributions to the NMR shifts are interpreted using relativistic density functional theory (DFT) calculations. The findings of this work bring important new information about the stability and transformations of Ru(III) compounds in aqueous solution and a recipe for decoding structural effects on paramagnetic NMR shifts. In the bigger picture, this work provides tight links between molecular structure and hyperfine effects useful for a range of transition-metal complexes.

## 2 Results and Discussion

### 2.1 Structure Characterization

Symmetric Ru(III) and Rh(III) coordination compounds **1**, **2**, **5**, and **6**, consisting of an octahedrally coordinated complex anion with two pyridine-based ligands in the axial positions, shown in Figure 1, were prepared as described in Section 4. The compounds were characterized by mass spectrometry, single-crystal X-ray diffraction, and NMR spectroscopy.

#### 2.1.1 Mass Spectrometry Analysis

**Figure 2:**
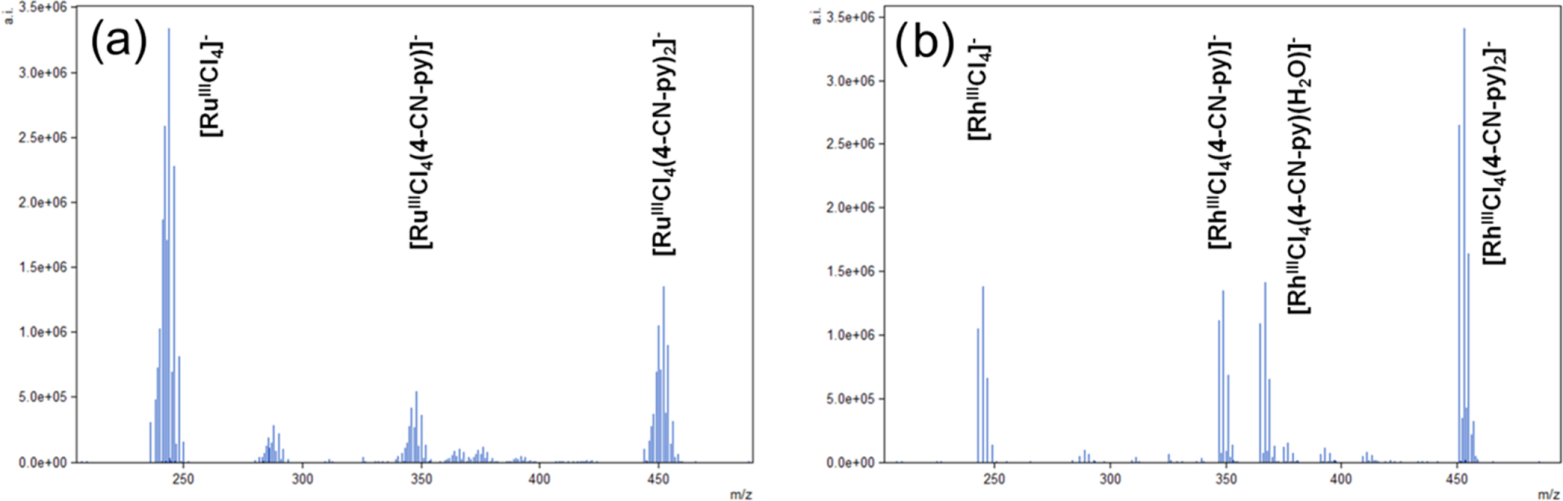
Portions of the ESI-MS (−) spectra of (a) Ru(III) compound PPh_4_^+^**1f** and (b) Rh(III) compound PPh_4_^+^**2f**.

The compounds were subjected to high-resolution electrospray ionization–mass spectrometry (ESI-MS) (−) analysis. Complex anions [*trans*-M^III^(L_eq_)_n_(4-R-pyridine)_2_]^−^ (M^III^ = Ru, Rh), (L_eq_ = Cl, *n* = 4; L_eq_ = ox, *n* = 2) were detected as molecular signals in the ESI-MS spectra (*m*/*z* value, isotopic profile) of compounds **1**, **2**, **5**, and **6** (for PPh_4_^+^ salts, see Table S1 and Figure S1). The signals observed at smaller *m*/*z* values correspond to anionic ionization fragments [M^III^Cl_4_]^−^, [M^III^Cl_4_(4-R-pyridine)]^−^, and [Ru^III^(ox)_2_]^−^ or their aqua forms. See Figure 2 for examples of compounds **1f** and **2f** (PPh_4_^+^salts).

#### 2.1.2 X-ray Diffraction Analysis

**Figure 3:**
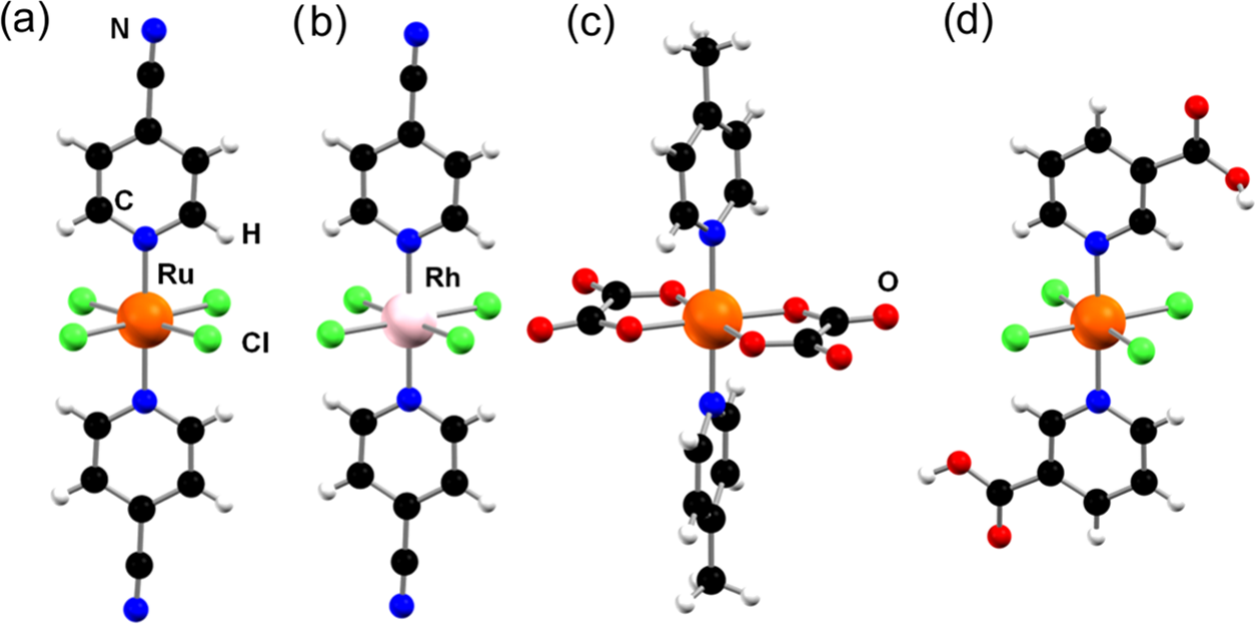
Molecular structures of the complex anions (a) **1f**, (b) **2f**, (c) **5a**, and (d) **6d** as determined by single-crystal X-ray diffraction. Organic cations and co-crystallized solvent molecules have been omitted for clarity (cf. Figure S2).

The molecular and crystal structures of compounds **1f**, **2f**, **5a** (all PPh_4_^+^ salts), and LH^+^**6d** were determined by single-crystal X-ray diffraction analysis. To the best of our knowledge, the X-ray structure of compound PPh_4_^*+*^**2f** is the first example of a symmetric rhodium complex of this type with a *trans* arrangement of pyridine-based ligands.^17^ All four compounds adopt an octahedral arrangement of coordinating ligands around the Ru or Rh center, with four chlorine atoms (**1f**, **2f**, and **6d**) or two chelating oxalate anions (**5a**) located in the equatorial plane and two 4-methylpyridine (**5a**), 3-carboxypyridine (**6d**), or 4-cyanopyridine (**1f** and **2f**) ligands in axial positions (Figure 3). For the crystal packing of PPh_4_ salts of **1f**, **2f**, **5a**, and LH^+^**6d**, see Figure S2.

The interatomic distance Ru—N obtained from the X-ray diffraction analysis of compound **1f** is somewhat longer compared with its Rh—N counterpart in **2f**. This difference reflects the different covalent radii of the two metals (Ru: 146 pm, Rh: 142 pm)^18^ but says nothing about the stability of the metal–ligand bond, see Section 2.2.

#### 2.1.3 NMR Analysis and Hyperfine Shifts

Q^+^[*trans*-Ru^III^Cl_4_(L_1_)(L_2_)] compounds are known to undergo aquation processes in a water environment—replacement of ligands in the coordination sphere of the ruthenium atom with water.^19−21^ Therefore, we started our analysis with NMR measurements in organic solvents (dimethylformamide-*d*_7_, DMF-*d*_7_, and dimethylsulfoxide-*d*_6_, DMSO-*d*_6_). To get a rough estimation of the temperature-independent orbital (δ_L_^orb^) and temperature-dependent hyperfine (δ_L_^HF^) contributions to the total NMR shifts of the paramagnetic compounds **1**, we performed a series of NMR measurements at various temperatures and extracted data from the resulting Curie plots^8,22−25^ (shown in Figure S3 in the Supporting Information). The experimental ^1^H and ^13^C NMR shifts for compounds **1** (LH^+^) and **5** (PPh_4_^+^)—which contain organic cations—dissolved in DMF-*d*_7_ are summarized in Table 1.

**Table 1:** Experimental NMR Shifts^a^ of Ligand Atoms L (δ_L_^tot^) and Their Orbital (δ_L_^orb^) and Hyperfine (δ_L_^HF^) Components^b^ for Symmetric Compounds LH^+^**1** and PPh_4_^+^**5** in DMF-*d*_7_ at 293 K in ppm^c^,^d^

Atom		**1a**	**1b**	**1c**	**1d**	**1e**	**1f**	**5a^e^**	**3a^f^**	**3f^f^**
H2	δ^orb^	+9.5	+10.0	+7.3	+8.7	+8.7	+9.1	+18.3	+6.6	+7.1
	**δ^HF^**	**–27.4**	**–26.8**	**–23.8**	**–22.2**	**–20.9**	**–20.6**	**–54.0**	**–14.0**	**–11.9**
	δ^tot^	–17.9	–16.8	–16.5	–13.5	–12.2	–11.5	–35.7	–7.4	–4.8
H3	δ^orb^	+8.4	+8.5	+8.7	+8.2	+8.4	+7.9	+10.4	+7.4	+7.6
	**δ^HF^**	**–15.1**	**–15.1**	**–14.8**	**–13.9**	**–13.5**	**–13.2**	**–16.1**	**–9.4**	**–8.5**
	δ^tot^	–6.7	–6.6	–6.1	–5.7	–5.2	–5.3	–5.7	–2.0	–0.9
										
C2	δ^orb^	+178	+177	+177	+173	+175	+172	+210	+162	+165
	δ^HF^	**–140**	**–137**	**–139**	**–134**	**–136**	**–135**	**–167**	**–72**	**–78**
	δ^tot^	+38	+40	+38	+39	+38	+37	+43	+90	+87
C3	δ^orb^	+117	+117	+115	+117	+114	+120	+118	+123	+124
	δ^HF^	**–23**	**–21**	**–20**	**–17**	**–17**	**–14**	**–9**	**–25**	**–17**
	δ^tot^	+95	+96	+95	+100	+96	+106	+109	+98	+107
C4	δ^orb^	+158	+144	+158	+144	+142	+123	+175	+152	+123
	δ^HF^	**–60**	**–58**	**–63**	**–61**	**–63**	**–65**	**–52**	**–27**	**–34**
	δ^tot^	+98	+86	+95	+83	+79	+58	+123	+125	+89
C5	δ^orb^	+21	–	+138	+167	+125	+118	+19	+21	+117
	δ^HF^	+1	–	**–2**	**+9**	**+8**	**+22**	**+1**	**0**	**+11**
	δ^tot^	+22	–	+136	+176	+133	+140	+20	+21	+128

a Referenced relative to the signal of the solvent (8.03 ppm for ^1^H; 163.2 for ^13^C).

b The precision of the analysis from the Curie plots is estimated to be about 0.5 and 3 ppm for the ^1^H and ^13^C NMR shifts, respectively; see ref (8).

c Previously published NMR shifts of selected asymmetric compounds LH^+^**3** are included for comparison, ref (8)

d For NMR shifts measured in DMSO-*d*_6_, see Table S2.

e δ_Cox_^orb^= 82 ppm, δ_Cox_^HF^ = −108 ppm, δ_Cox_^tot^ = −26 ppm.

f Data from ref (8).

#### 2.1.4 ^1^H NMR Spectroscopy in Organic Solvents

We obtained very good agreement between the ^1^H NMR shifts of atoms of the 4-R-pyridine ligands in diamagnetic Rh(III) analogs (e.g., H2: 9–10 ppm, H3: 7–8 ppm in Table S2) and the orbital contributions to the ligand hyperfine shifts of paramagnetic Ru(III) compounds (H2: 8–10 ppm, H3: 8–9 ppm in Table 1). However, the total ^1^H NMR signals in compounds **1** are additionally influenced by the hyperfine interaction, which results in a substantial shielding of the ligand nuclei in compounds **1** with respect to those in the diamagnetic analogs **2** (not included in Table 1, see Table S2 in the Supporting Information to compare the total NMR shifts measured in DMSO-*d*_6_).

**Figure 4:**
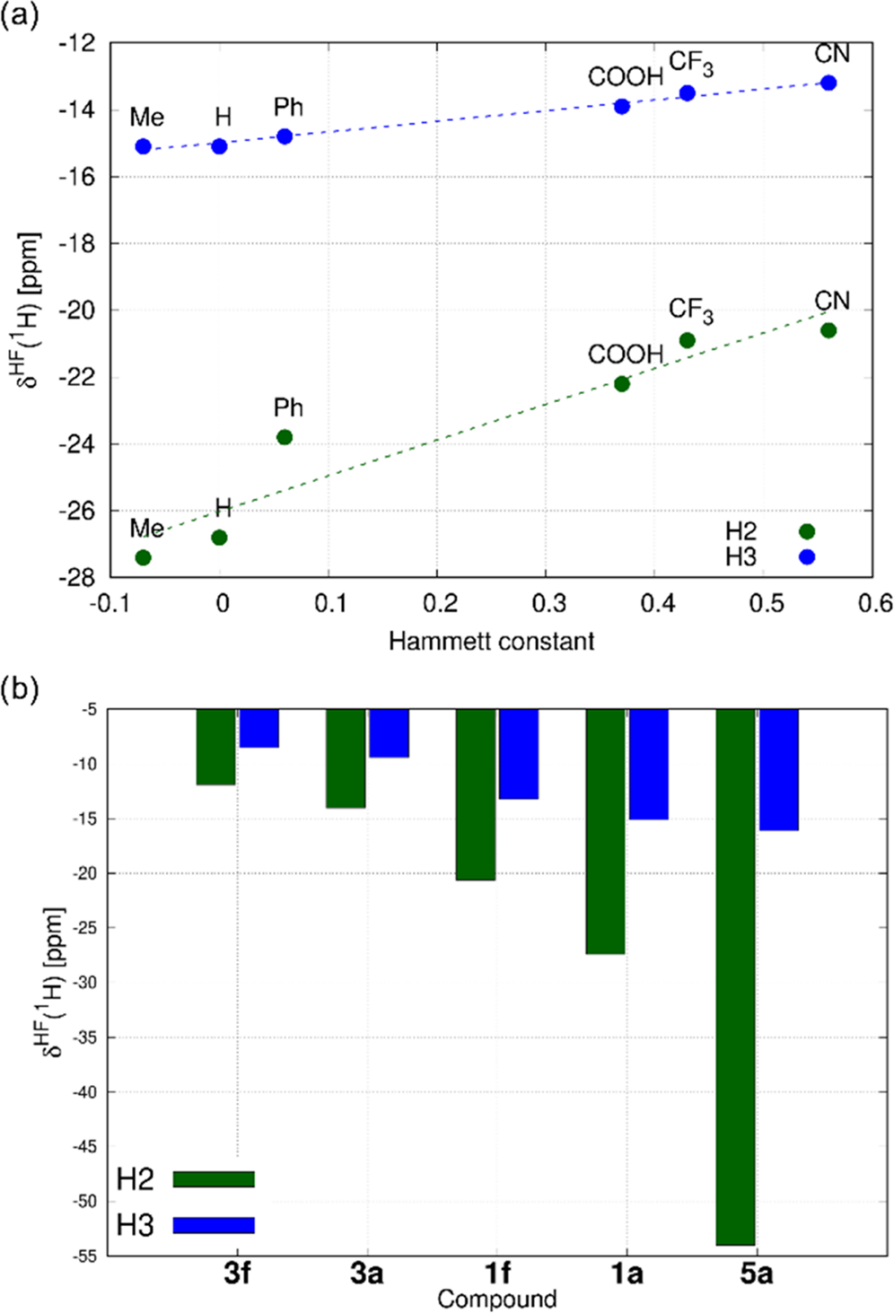
(a) Correlation of the experimental hyperfine ^1^H NMR shifts of atoms H2 and H3 in compounds **1a**–**1f** with the Hammett constant^26^ of substituent R. (b) Effect of the *trans* ligand (**1** vs **3**) and equatorial ligands (**1a** vs **5a**) on the experimental hyperfine ^1^H NMR shifts of atoms H2 and H3. The NMR spectra were measured in DMF-*d*_7_ and the hyperfine NMR shifts are reported at 293 K.

The hyperfine shift of H2 is systematically reduced from −27.4 ppm for compound **1a**, with substituent R (CH_3_) having a positive inductive effect, down to −20.6 ppm for compound **1f**, with the negative inductive effect^26^ and polarizable π-space of substituent R (CN), Figure 4a. This trend is in line with our observations for asymmetric complexes Q^+^[*trans*-Ru^III^Cl_4_L(DMSO-*S*)]^−^ reported previously (compounds **3a** vs. **3f** in Table 1).^8^ Similarly, the magnitude of the hyperfine shift of the H3 atom is reduced from −15.1 ppm (**1a**, CH_3_) to −13.2 ppm (**1f**, CN).

Comparing hyperfine ^1^H NMR shifts of both H2 and H3 in the symmetric compounds **1** with those in the asymmetric analogs **3** (e.g., **1a** vs **3a**), we identified significantly more negative values for compounds **1** as shown in Figure 4b. The hyperfine effects are even larger in compounds **5**, where four equatorial chlorides are replaced by two bidentate oxalate ligands (e.g., **1a** vs **5a**). The electronic origins of these ligand-induced NMR trends are analyzed using DFT calculations and discussed in Section 2.3.

#### 2.1.5 ^13^C NMR Spectroscopy in Organic Solvents

**Figure 5:**
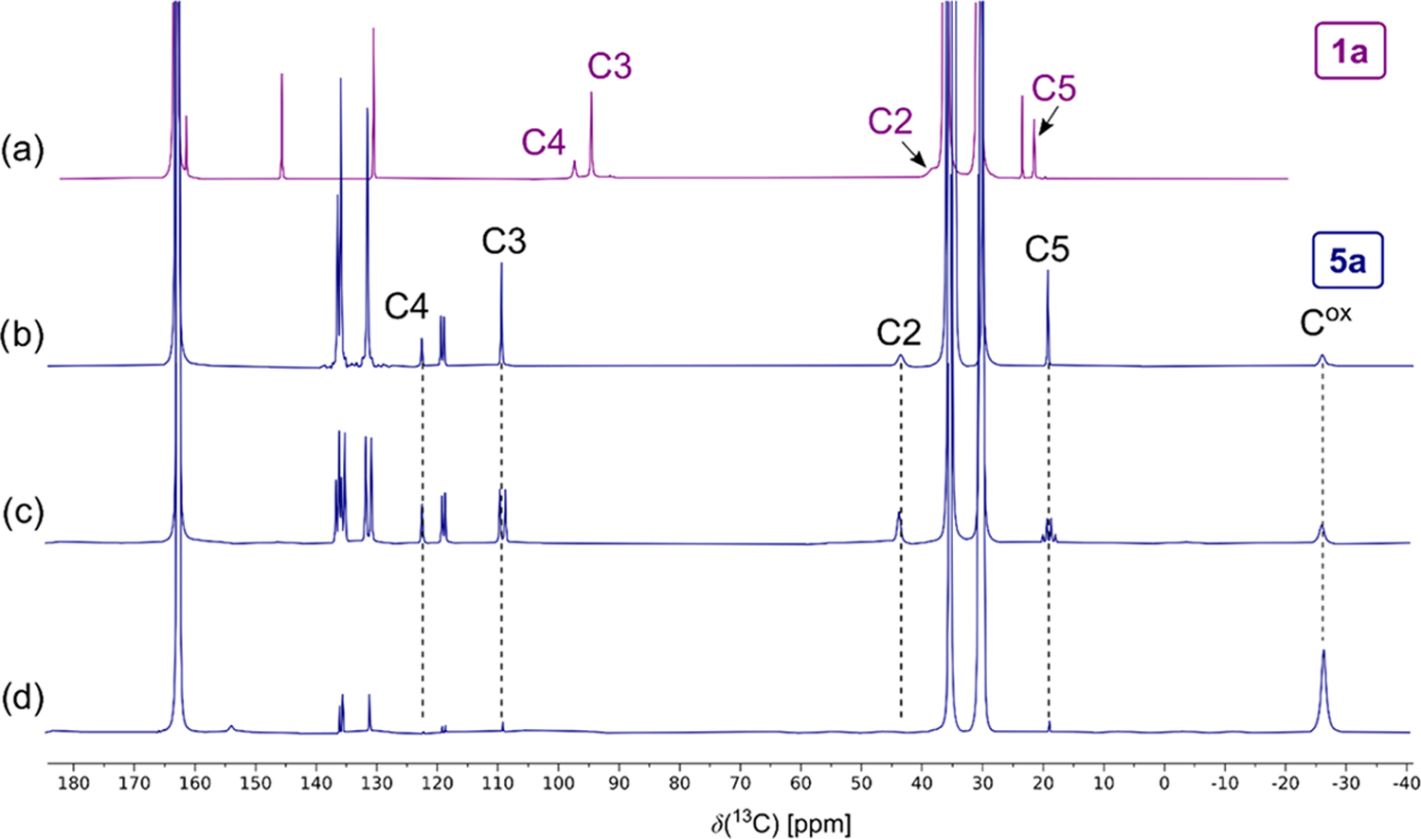
^13^C{^1^H} NMR spectra of (a) compound LH^+^**1a** and (b) compound PPh_4_^+^**5a**. (c) ^13^C{^1^H2-decoupled} NMR spectrum of compound PPh_4_^+^**5a**. (d) ^13^C{^1^H} NMR spectrum of compound PPh_4_^+^**5a** with selective ^13^C labeling of the oxalate carbons. All ^13^C NMR spectra were recorded in DMF-*d*_7_ at 293 K.

The ^13^C NMR signals for compounds **1** and **5a** have been assigned according to their characteristic paramagnetic NMR shifts, paramagnetic-relaxation broadening, and indirect single-bond ^1^H–^13^C couplings. As examples, the ^13^C{^1^H} NMR spectra of compounds **1a** and **5a** measured in dimethylformamide-*d*_7_ are shown in Figure 5a,b, respectively. The signals were unequivocally assigned based on the presence of characteristic ^1^H–^13^C splittings (doublet for aromatic C3, quartet for methyl C5; e.g., see compound **5a** in Figure 5c) in ^1^H-coupled ^13^C NMR spectra.

However, for compound **5a**, the assignment of two ^13^C NMR resonances, at +43 and −26 ppm, to the atoms C2 and oxalate was ambiguous because none of the fine splitting expected from the indirect ^1^H2–^13^C2 interaction was observed. The resonance at +43 ppm was tentatively assigned to the atom C2 based on a slight sharpening of the resonance in the selectively {^1^H2}-decoupled ^13^C NMR spectrum shown in Figure 5c. To confirm this assignment unequivocally, we synthesized compound **5a** with ^13^C selectively enriched oxalate giving the ^13^C NMR spectrum shown in Figure 5d.

**Figure 6:**
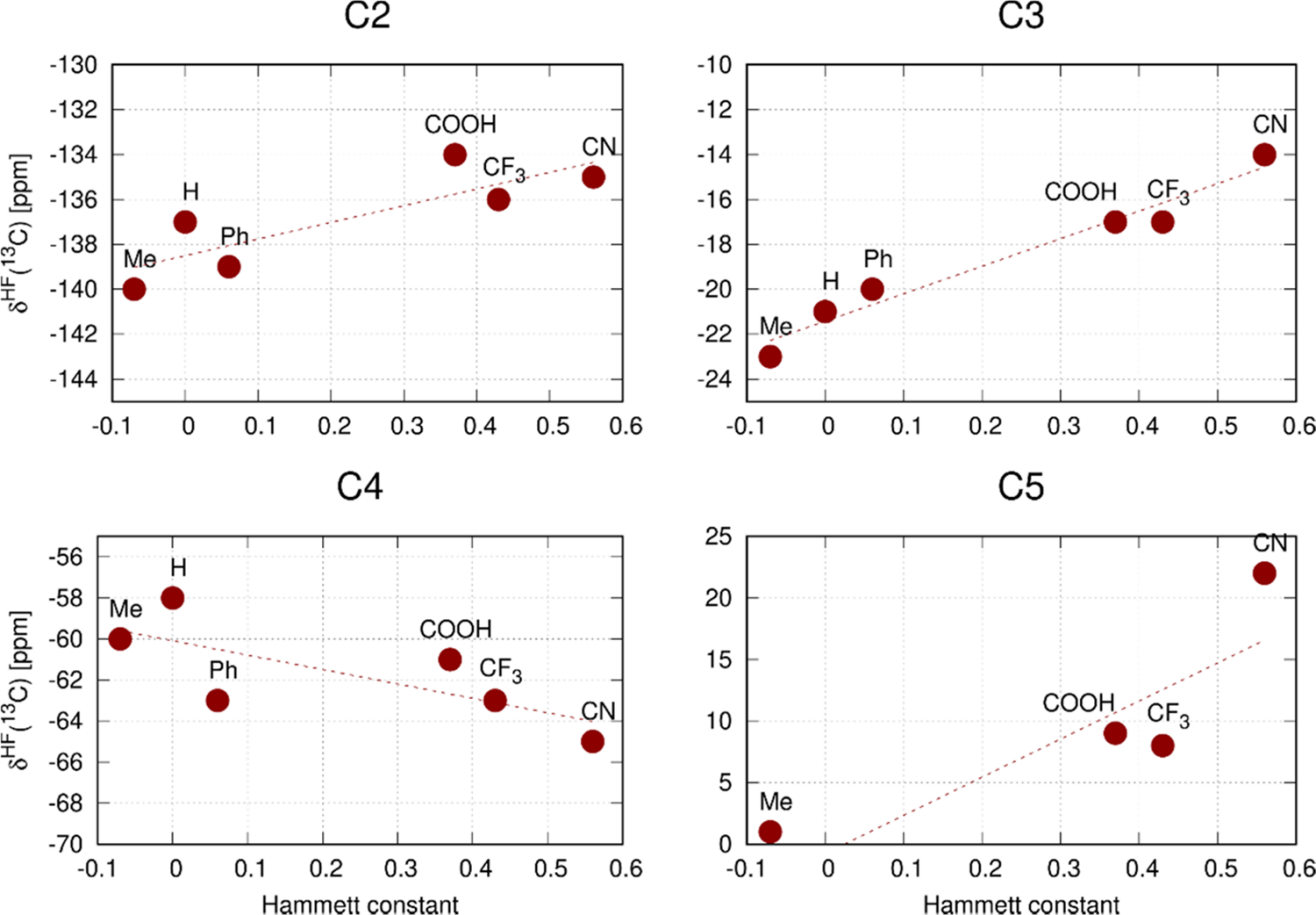
Correlation of the hyperfine ^13^C NMR shifts (DMF-*d*_7_ at 293 K) of atoms C2–C5 in compounds **1a**–**1f** with the Hammett constant^26^ of substituent R.

The characteristic NMR linewidths related to the distance of the individual atoms from the paramagnetic center and, especially, the distribution of spin density resulting from the spin delocalization and polarization of the pyridine ligands (cf. C2, C3, and C4 in Figure 5) have been analyzed and reported for asymmetric compounds **3** previously.^8,9^ The substituent-induced^26^ trends in the hyperfine ^13^C NMR shifts summarized in Table 1 are graphically shown in Figure 6. The effect of substituent R on pulling(pushing) electrons from(to) the pyridine ring and the spin polarization of individual atoms of the pyridine ligand are interpreted in Section 2.3.

#### 2.1.6 ^1^H NMR Spectroscopy in Water

**Figure 7:**
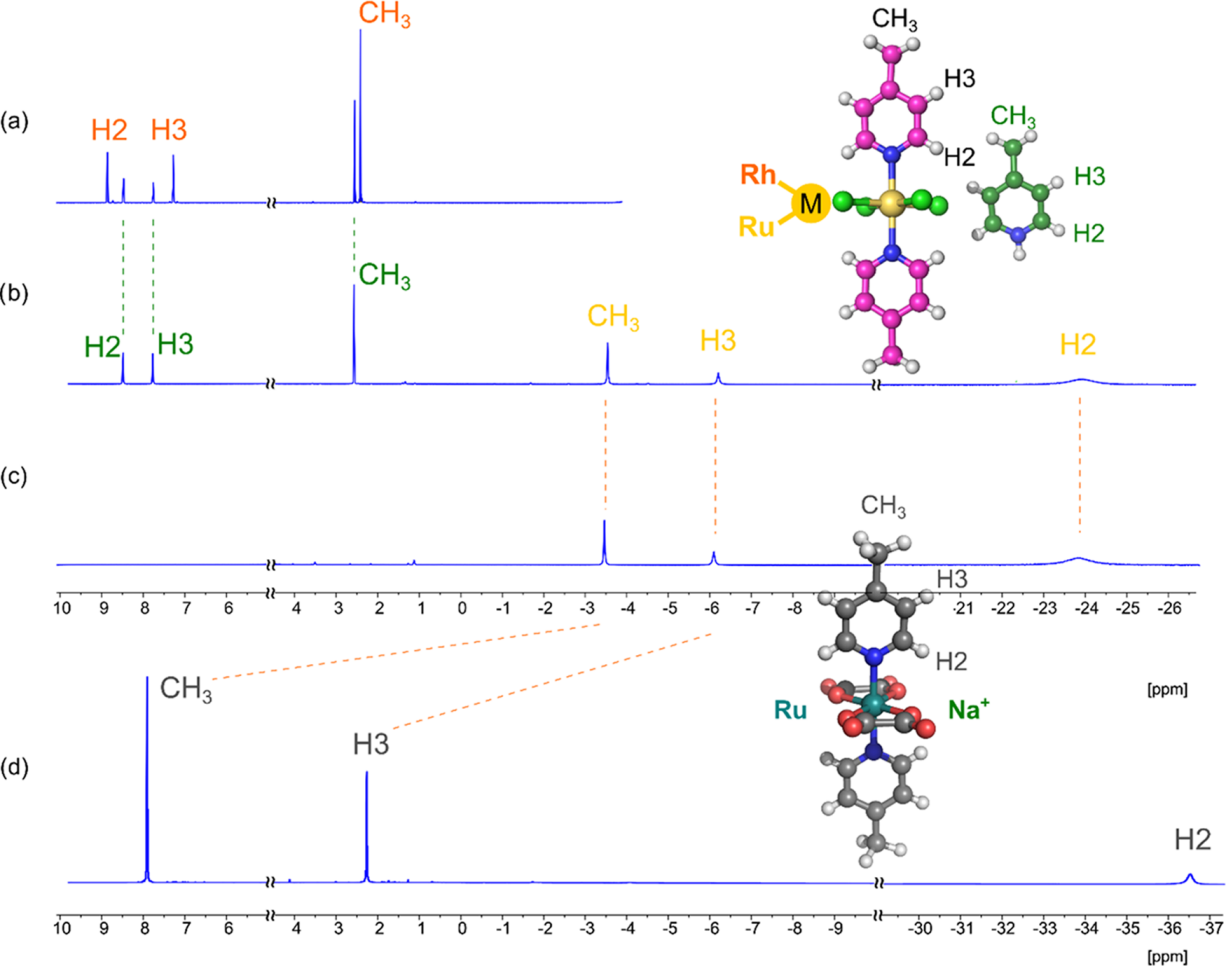
^1^H NMR spectra of (a) Rh(III) compound L_1_H^+^**2a**, (b) Ru(III) compound L_1_H^+^**1a**, (c) Ru(III) compound K^+^**1a**, and (d) Ru(III) compound Na^+^**5a** measured in D_2_O at 298 K.

Because of the biological importance of the symmetric compounds **1**,^27,28^ we also investigated their structures in aqueous environment (D_2_O). The NMR behavior we observed in water was qualitatively similar to that in organic solvents, including the large effects of equatorial ligands (see complex anions K^+^**1a** and Na^+^**5a** in Figure 7). The ^1^H NMR shifts of nuclei belonging to the organic cation (LH^+^) proved to be almost independent of the nature of the complex anion (e.g., see **2a** in Figure 7a vs **1a** in Figure 7b). Similarly, the NMR shifts for the complex anion **1a** in solution are almost independent of the nature of the counter-cation (cf. LH^+^**1a** and K^+^**1a** in Figure 7b,c, respectively). This indicates relatively weak association of cations with anions in water (vide infra).

Comparison of the NMR shifts measured in organic solvents and water showed very large solvent effects on the NMR resonances particularly for compound **5a**. This effect is further analyzed and discussed in Section 2.3.

### 2.2 Stability of Metal–Ligand Bond in Water

It is well known that spontaneous structural transformations in aqueous solutions are essential to the biological response to many transition-metal-based prodrugs, including compounds **1**–**4**. Therefore, we investigated the structural stability of representative compounds **1a** and **2a** (especially the integrity of the M–N bond) using ^1^H NMR experiments under various conditions. Solvent nucleophiles (H_2_O or OH^–^) can, in principle, replace either chloride (equatorial ligand) or pyridine (axial ligand),^29^ see structures in Figure 1. When H_2_O is substituted for Cl^–^ in compound **1a**, the charge of the complex is changed from −1 to 0, but the axial organic ligand (4-Me-Py) is affected only indirectly by a modulation of the paramagnetic NMR effects propagated from the paramagnetic center toward the pyridine moiety.^8,9,30,31^ In the case of pyridine, the axial 4-Me-Py ligand is dissociated from the ruthenium center as manifested by the appearance of NMR resonances belonging to a diamagnetic form of the 4-Me-Py ligand.

**Figure 8:**
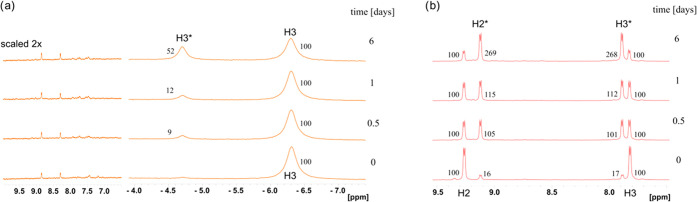
^1^H NMR spectra of (a) K^+^**1a** and (b) K^+^**2a** measured immediately after the sample preparation (1 mM in D_2_O, 298 K) and after 0.5, 1, and 6 days of sample incubation at room temperature. The NMR resonances of newly appearing hydrolyzed species (see also Table S3 in the Supporting Information) are marked by asterisks.

We have proven that the dominating mechanism of aquation for compound **1a** is the splitting off of Cl^–^ because the newly appearing NMR resonances of the 4-Me-Py ligand (Figure 8a) remain paramagnetically shielded over time. No change in the diamagnetic NMR region of aromatic hydrogen atoms (8–9 ppm) was observed even after 6 days of incubation in D_2_O at laboratory temperature. The half-life of **1a** in the original chemical constitution *trans*-[RuCl_4_L_2_]^−^ is estimated to exceed 6 days, which is notably longer than what has been observed for imidazole analogs (∼3 days).^29^ Note in passing that only slight precipitation was observed in our experiments in contrast to the behavior reported for Na^+^[*trans*-RuCl_4_(ind)_2_]^−^.^32^

The process of hydrolysis in D_2_O (aquation) is significantly faster for rhodium(III) compounds **2**, as indicated, for example, for compound K^+^**2a** in Figure 8b. Conversion of 50% of **2a** to **2a_aq** in D_2_O is estimated from the NMR experiments to require only about 12 h. In parallel to compound **1a**, this hydrolysis involves splitting the Rh–Cl bond, as has been confirmed by adding 4-R-pyridine base to the NMR sample, see Figure S4 in the Supporting Information.

We also investigated the effects of concentration and environment on the rate of hydrolysis of the M–Cl bond. NMR samples with the higher 5 mM concentration of metallocomplex in solution hydrolyzed faster than the corresponding 1 mM solution, probably because of autocatalysis and the formation of (hydro)oxo bridges^33,34^ (Figure S5 in the Supporting Information).

The study of the stability of symmetric complexes **1a** in water was further extended by an analogous investigation of asymmetric compounds represented by complex **3d**. In contrast to the aquation of **1a**, the aquation process of **3d** affects primarily the axial ligands, as evidenced by the gradual appearance of NMR resonances corresponding to the free ligands (Figure S6). The fastest process is the hydrolysis of DMSO, with the signal intensity of free 4-COOH-Py increasing more slowly. This is in agreement with observations made previously by Webb et al.^35^ Some minor forms also occur in the NMR spectra, suggesting the possible hydrolysis of chloride(s).

The difference in the stability of the Ru–N bond in compounds **1a** and **3a** can be rationalized by considering the kinetic *trans*-effect of the attached ligands.^36,37^ In the symmetric complex **1a**, equatorial chlorides exert a larger *trans*-effect than 4-COOH-Py and the mono-hydrolyzed product is formed by the exchange of an equatorial chloride. In contrast, the 4-COOH-Py ligand can be replaced by water in the asymmetric complex **3d**, where an axial DMSO ligand with a large *trans*-effect (vide infra) is present (though hydrolysis of the Ru–S bond is faster).

**Figure 9:**
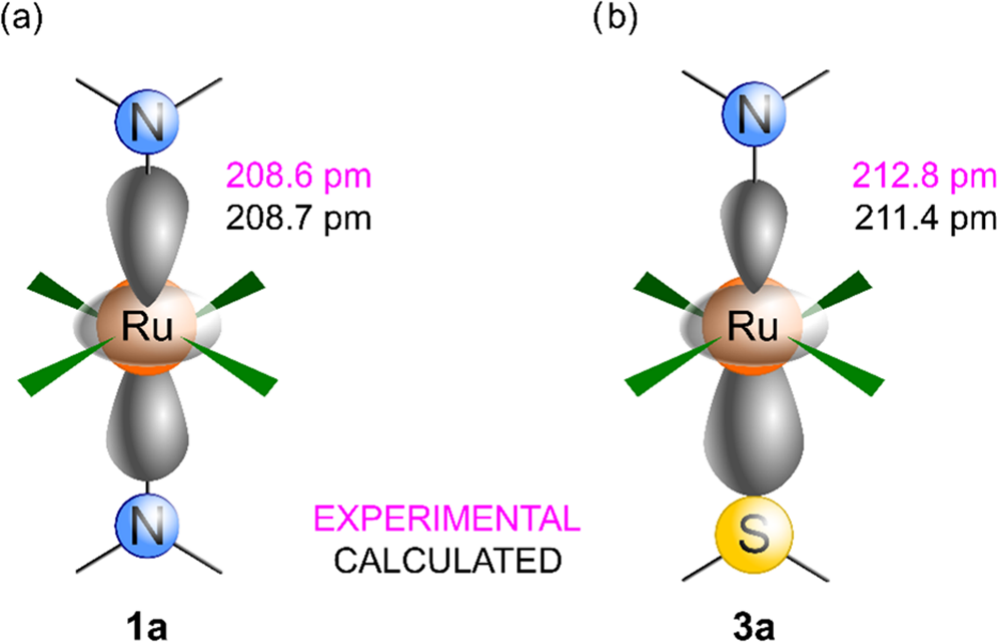
Schematic representation of the Ru–N bond in (a) symmetric compound **1a** and (b) asymmetric compound **3a**, as optimized at the scalar-relativistic DFT level (PBE0/def2-TZVPP/COSMO-DMF) in the program Turbomole^38^ (black) and obtained from X-ray diffraction analysis (magenta). For the analogous data obtained for Rh(III) compounds, see Figure S7 in the Supporting Information.

The difference in reactivity of the Ru–N bond in **1a** and **3a** is accompanied by structural differences observed in crystal structures. These were reproduced in geometry optimizations of the molecular models at the scalar-relativistic DFT level (see Section 4, Methods). The Ru–N bond distances obtained from X-ray diffraction analysis and DFT-optimized geometries are compared in Figure 9.

The Ru–N bond distance is slightly shorter in compound **1a** and its elongation in compound **3a** (due to the structural *trans*-effect of the DMSO ligand)^37^ is assumed to make it somewhat weaker (see estimation from the energy decomposition analysis of the Ru–N bond in Table S4). This structural change can be indirectly linked with a higher susceptibility of the Ru–N bond to breaking (kinetic *trans*-effect),^37^ as experimentally identified and discussed above. However, the slight modulation of the Ru–N bond distance has a large effect on the propagation of spin density from the ruthenium center toward the ligand atoms, which contributes to the hyperfine NMR shifts as discussed in the following section.

### 2.3 Relativistic DFT Calculation and Interpretation of Hyperfine NMR Shifts

The analysis of the experimental NMR shifts introduced in Section 2.1 revealed a substantial dependence of the hyperfine shifts on the nature of (i) the 4-R substituent of the pyridine, (ii) the substituent in the *trans* position at the Ru(III) center, and (iii) the nature of the equatorial ligands. To interpret the trends observed in the NMR shifts (Figures 4 and 6), we performed relativistic DFT calculations of the EPR parameters (electronic **g**-tensor, hyperfine coupling **A**-tensor; for MO diagram, see Figure S8) and, subsequently, the NMR shifts according to eqs 1 and 2.^16^
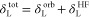
1
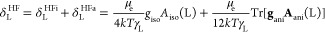
2where δ_L_^HFi^ (derived from the isotropic *g* and *A*) and δ_L_^HFa^ (derived from the **g** and **A** anisotropy)^9^ contain the traditional contact and pseudocontact contributions, respectively.^12,13,39^ Here, *kT* represents the thermal energy, μ_e_ is the Bohr magneton, and γ_L_ is the gyromagnetic ratio of nucleus L. The data for representative compounds **1a**, **3a**, and **5a** are summarized in Table 2.

**Table 2:** Experimental (δ_exp_^HF^, in DMF-*d*_7_) and Calculated (δ_cal_^HF^, SO-ZORA/PBE0/TZ2P/COSMO-DMF) Hyperfine Contributions to the NMR Shifts (in ppm) for the Symmetric Compound **1a**, Its Asymmetric Analog **3a**,^8^ and Compound **5a** at 293 K^a^,^b^

	**1a**			**3a**			**5a**			**5a·2H**_**2**_**O**	
	**δ_exp_^HF^**	δ_cal_^HF^	δ_cal_^HFi^/δ_cal_^HFa^	**δ_exp_^HF^**	δ_cal_^HF^	δ_cal_^HFi^/δ_cal_^HFa^	**δ_exp_^HF^**	δ_cal_^HF^	δ_cal_^HFi^/δ_cal_^HFa^	δ_cal_^HF^	δ_cal_^HFi^/δ_cal_^HFa^
H2	**–27.4**	–21.2	–4.4/–**16.8**	**–14.0**	–14.7	–6.8/**–7.9**	**–54.0**	–20.1	+0.4/–**20.5**	–26.5	–8.2/–18.3
H3	**–15.1**	–14.4	**–13.8**/–0.6	**–9.4**	–9.7	**–9.9**/+0.3	**–16.1**	–16.6	**–14.0**/–2.6	–13.7	–10.4/–3.2
H5	**–10.6**	–10.9	**–9.7**/–1.3	**–6.0**	–6.1	**–5.2**/–0.9	**–9.0**	–11.7	**–9.7**/–2.0	–8.1	–6.0/–2.1
											
C2	**–140**	–140	–130/–10	**–72**	–75	–74/–1	**–167**	–190	–163/–27	–140	–158/+18
C3	**–23**	–21	–20/–1	**–25**	–27	–25/–2	**–9**	+17	+17/0	+11	+16/–5
C4	**–60**	–60	–51/–9	**–27**	–29	–25/–4	**–52**	–59	–45/–14	–43	–32/–11
C5	**+1**	+7	+9/–2	**0**	+2	+3/–1	**+1**	+12	+15/–3	+8	+10/–2
Cox	–	–	–	–	–	–	**–108**	–246	–294/+48	–271	–310/+38

a The calculated values of the hyperfine NMR shifts are decomposed into isotropic contributions originating in isotropic (δ_cal_^HFi^) and anisotropic (δ_cal_^HFa^) hyperfine interactions.^9^

b For NMR shifts calculated in an implicit model of water, see Table S6 in the Supporting Information.

#### 2.3.1 Effect of Substituent R on Pyridine

**Figure 10:**
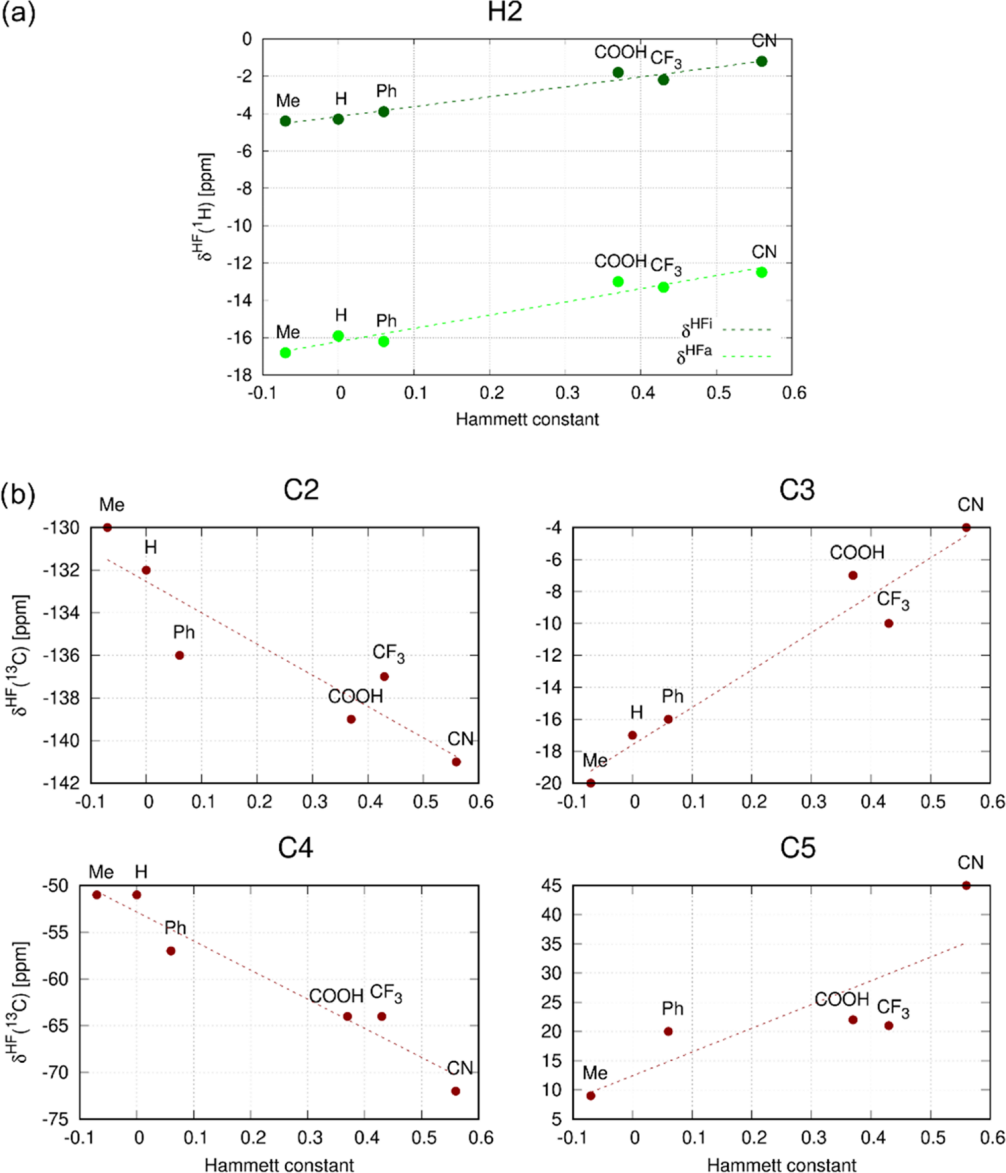
Correlation of the DFT-calculated (a) δ_L_^HFi^ and δ_L_^HFa^ for atom H2, and (b) δ_L_^HFi^ for atoms C2–C5 in compounds **1a**–**1f** with the Hammett constant of substituent R.

The calculated δ_L_^HFi^ and δ_L_^HFa^ contributions to the isotropic hyperfine NMR shifts for all hydrogen and carbon atoms in compounds **1a**–**1f** are summarized in Table S5. Correlations of the calculated contributions δ_L_^HFi^ and δ_L_^HFa^ for atom H2 and δ_L_^HFi^ for atoms C2–C5 in compounds **1a**–**1f** with the Hammett constants are shown in Figure 10.

The substituent effects (expressed by the Hammett constant of substituent R) on the calculated δ^HF^ for atom H3 are vanishingly small and will not be discussed further. The hyperfine NMR shift of the atom H2 is quite complex. As identified from the theoretical calculations, it is dominated by δ^HFa^ (−16.8 ppm for **1a** vs −12.5 ppm for **1f**, Table S5) because of the quite small Fermi-contact (FC) term and the partly compensating contribution of the paramagnetic spin-orbit (PSO) term (Table S7). The difference between **1a** and **1f** is given by the inverted sign of the FC term (in δ^HFi^) and the different negative contributions of the PSO and SD terms (Table S7b in the Supporting Information).

Trends in the experimental hyperfine ^13^C NMR shifts are qualitatively reproduced by calculated values for the atoms C3–C5 (cf. Figures 6 and 10). In all cases, the electronic effect of substituent R governs the spin polarization of the carbon atoms and the contact hyperfine shifts (δ^HFi^). However, the weak substituent-induced modulation of experimental δ^HF^ for atom C2 is opposite to that observed for our theoretical models. We assume that this discrepancy for C2 with a quite complex hyperfine mechanism (σ vs π hyperfine pathways)^8^ could be caused by the use of an inappropriate model of solvent that results in insufficient spin polarization of the equatorial ligands and overestimates the polarization of the axial pyridine ligands. This hypothesis is supported by the overestimation of some theoretical hyperfine ^13^C NMR shifts in a series of compounds **1**. For the effects of a simple explicit solvent, see below.

#### 2.3.2 Effect of Trans Ligand

**Figure 11:**
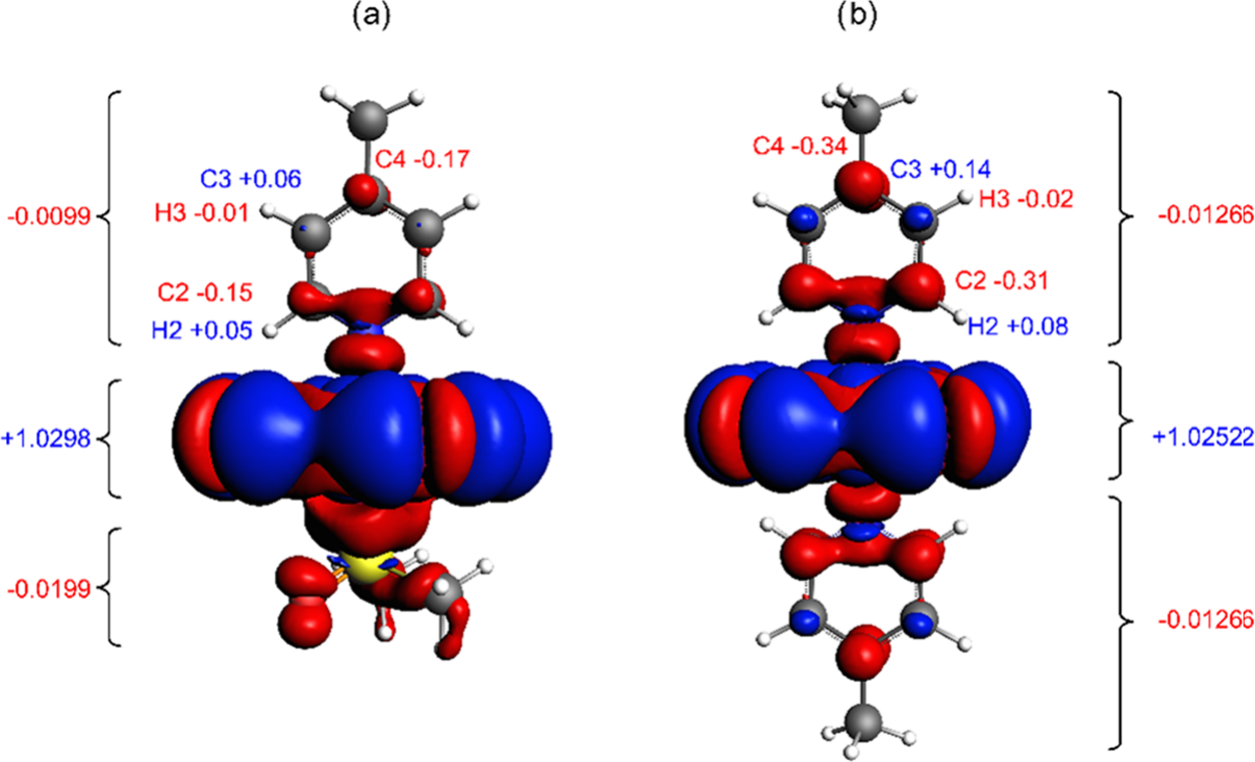
Distribution of spin density (cutoff ±10^–4^ au) calculated for (a) compound **3a** and (b) compound **1a** (ZORA/PBE0/TZ2P/COSMO-DMF, see Section 4.2). The numbers attached to the ligand atoms refer to the individual atomic spin populations multiplied by 10^2^. The values at side clips denote the spin population of the corresponding molecular fragment (4-MePy, [RuCl_4_], and DMSO).

Analysis of the experimental NMR shifts introduced in Section 2.1 revealed an increased magnitude of the hyperfine effects on the ^1^H and ^13^C resonances of the nuclei of the axial ligand in symmetric compounds **1** compared to those in their asymmetric analogs **3**.^8^ For example, the hyperfine shifts of atoms H3, C2, and C4 are approximately 1.5- to 2.0-fold larger in compounds **1** than in compounds **3** (Table 1). The increase in δ^HF^ of these atoms in compounds **1** is associated mainly with the δ^HFi^ contribution (Table S6 in the Supporting Information), which indicates a more efficient propagation of the spin density toward the ligand moiety in the symmetric systems. This correlates with shortening of the Ru–N bond in compound **1**, as identified and discussed above (Figure 9). The calculated distribution of the spin density and the atomic and fragment spin populations for compounds **1a** and **3a** are compared in Figure 11. The difference in C2 atomic spin populations between **1a** (−0.31 × 10^–2^) and **3a** (−0.15 × 10^–2^) is reflected in the difference between the calculated hyperfine values for this atom (−140 ppm for **1a** vs −75 ppm for **3a**). A similar pattern is also observed for atom C4, whereas the hyperfine shielding of C3 is relatively small and similar for **1a** and **3a** because of mutually compensating contributions from the σ- and π-polarization pathways.^8^

A somewhat more complex situation has been identified again for the atom H2, the hyperfine shift of which is dominated by δ^HFa^. This contribution is also largely modulated by the trans ligand (−16.8 ppm for **1a** vs −7.9 ppm for **3a** in Table 2). As identified from the analysis of individual hyperfine mechanisms (Table S7 in the Supporting Information), the larger value for compound **1a** results from the product of the larger SD term of hyperfine coupling **A** and the larger anisotropy of the **g**-tensor. This sensitivity of H2 (δ^HFa^) is obvious because of its very short distance from the paramagnetic center at the ruthenium atom, see Figure 11.

In summary, comparing the symmetric compound **1** with its asymmetric analog **3**, the structural *trans*-effect of the DMSO ligand on the Ru–N bond in compound **3** results in less efficient propagation of spin density to the *trans* pyridine, which, in synergy with a slightly smaller anisotropy of magnetization on the ruthenium center, results in diminished hyperfine contributions to the NMR shifts of the pyridine ligand atoms.

#### 2.3.3 Effects of Equatorial Ligands and Environment

**Figure 12:**
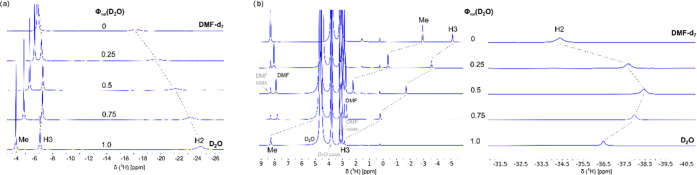
^1^H NMR spectra of (a) Na^+^**1a** and (b) Na^+^**5a** in DMF-*d*_7_ (top), D_2_O (bottom), and DMF-*d*_7_/D_2_O mixture (middle, volume fraction of D_2_O, ϕ_vol._ = 0.25, 0.50, and 0.75). All NMR measurements were performed at 298 K.

The experimental NMR shifts for compounds **1a** and **5a** shown in Table 1 indicate enhanced hyperfine effects on atoms of the pyridine ligand induced by substituting two oxalates for the four chlorides coordinated to the ruthenium atom in the equatorial plane. To interpret the differences in the experimental NMR resonances between **1a** and **5a**, we again performed DFT calculations of the NMR shifts. However, the calculated data deviate from the experimental values significantly (e.g., H2, C2, and C^ox^ for **5a** in Table 2). This can be due, in part, to the insufficient DFT description of the oxygen-based equatorial ligands and spin distribution in compound **5a**. Further, we hypothesize that the other part of the observed deviations is caused by an insufficient solvent model and specific solute-solvent interactions missing from our theoretical calculations using an implicit solvent model (vide supra). To support this hypothesis, we performed additional ^1^H NMR experiments for compounds **1a** and **5a** (both Na^+^ salts) to examine the effect of solvent composition (volume fraction of D_2_O in the mixture with DMF-*d*_7_, ϕ_vol._ = 0–1) on the ^1^H NMR resonances of atoms of the 4-Me-Py ligand, Figure 12 (for the NMR shifts, see Table S8 in the Supporting Information).

**Figure 13:**
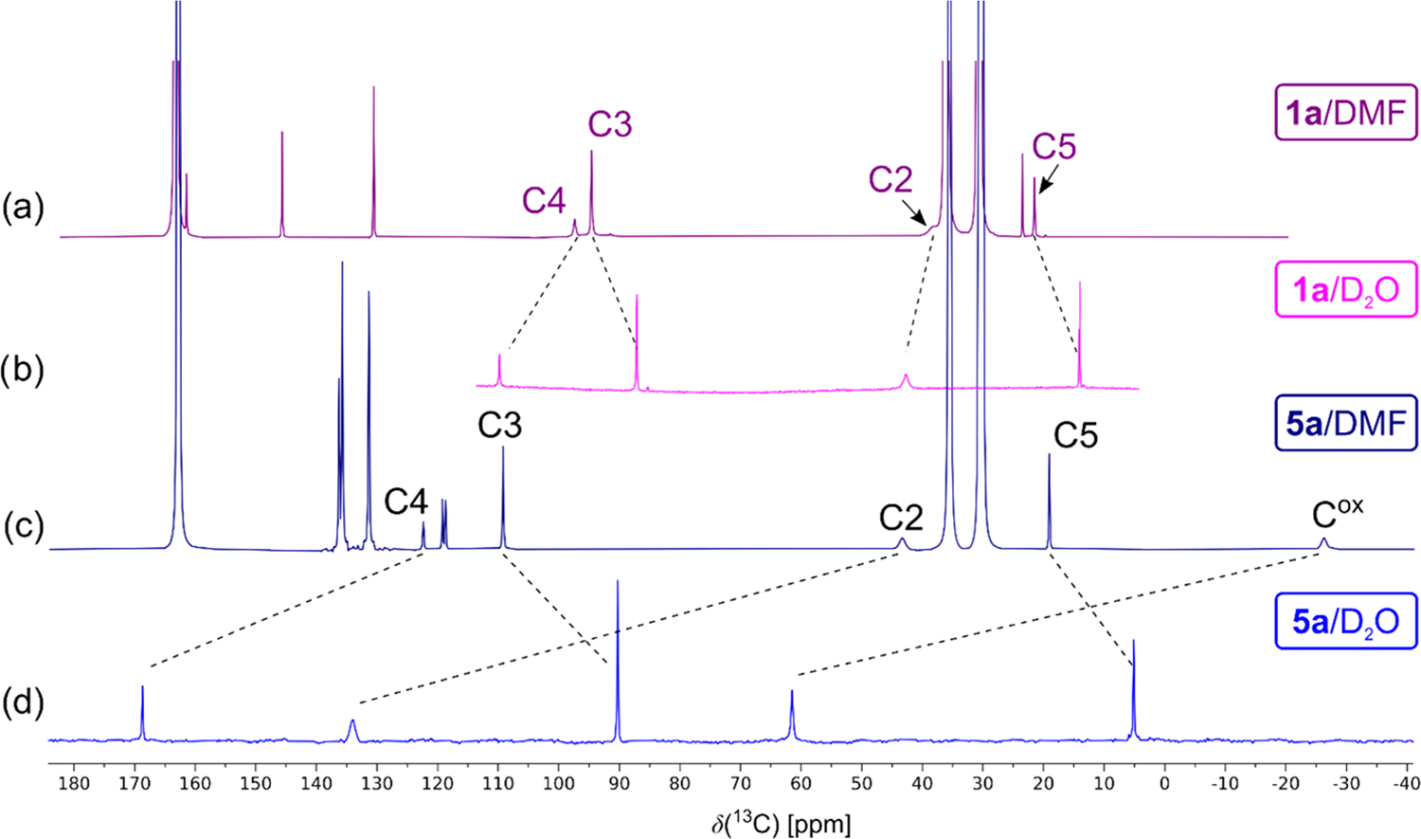
^13^C{^1^H} NMR spectrum of (a) compound LH^+^**1a** in DMF-*d*_7_, (b) compound Na^+^**1a** in D_2_O, (c) compound PPh_4_^+^**5a** in DMF-*d*_7_, and (d) compound Na^+^**5a** in D_2_O. All NMR spectra were recorded at 293 K.

A large effect of solute-solvent interactions on the NMR shifts was also detected for ^13^C signals, compared in DMF-*d*_7_ and D_2_O in Figure 13. Solvent effects of less than 20 ppm (C2 and C4 deshielding, C3 and C5 shielding) were obtained for compound **1a** with a RuCl_4_ core. Although the same polarization pattern was observed for compound **5a**, the solvent-induced NMR shift perturbations are substantially larger (e.g., effects on C2 and C^ox^ amount to approximately +90 ppm). This is clear evidence of the enormous response of the paramagnetic ruthenium center with oxalate ligands to its environment (solvent).

**Figure 14:**
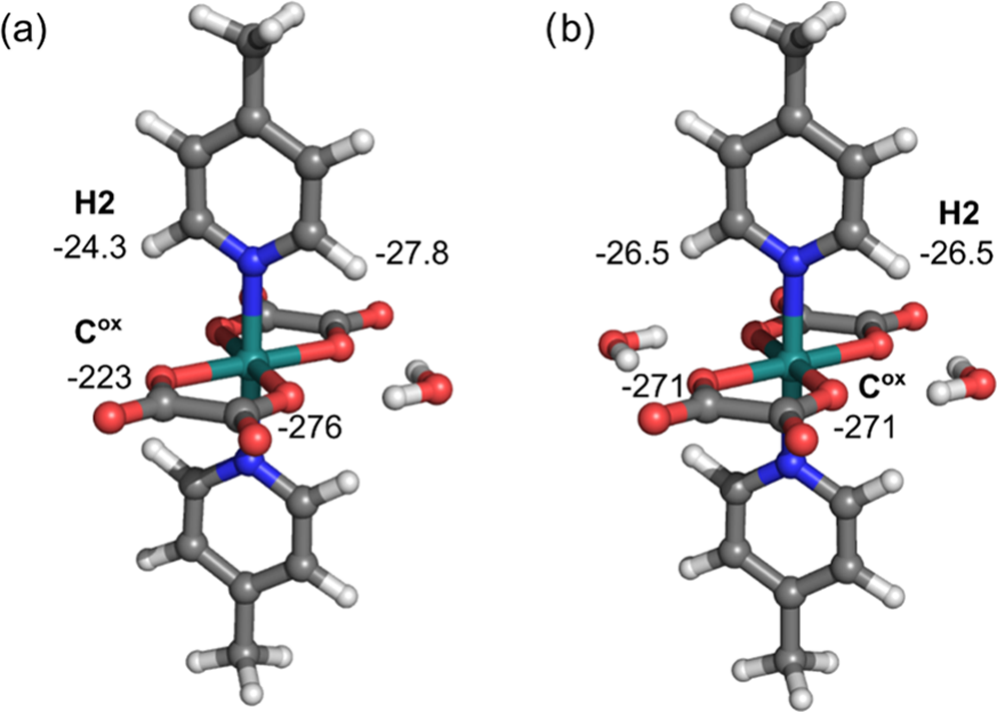
DFT-optimized structures of simple supramolecular assemblies of (a) H_2_O and (b) 2H_2_O coordinated to the oxygen atoms of both oxalates in compound **5a**. Numbers shown next to H2 and C^ox^ indicate the calculated hyperfine shifts of the corresponding atoms.

The perturbations of the NMR shifts caused by changing the solvent indicate important solute-solvent interactions, which are more obvious for compound **5a**. Therefore, we constructed a simple model of a water molecule hydrogen-bonded to two coordinated oxygens—one from each of the two oxalates in **5a** (Figure 14a)—and calculated the NMR shifts for the resulting supramolecular assembly. No consistent improvement in the theoretical NMR data relative to the experimental values was achieved (Table 2), but the observed effects confirm the importance of the supramolecular interactions. Note in passing that analogous large effects of the intermolecular interactions on the hyperfine NMR shifts have been reported for a crystal environment.^40^ To further explore the role of intermolecular contacts and rationalize the discrepancy between the calculated and experimental values, we modeled **5a** with two coordinated water molecules (**5a**·2H_2_O, Figure 14b). The role of the second water molecule is in line with that obtained for **5a**·H_2_O and further slightly enhances the averaged hyperfine shifts of equivalent atoms.

In summary, our results clearly show the important effects of molecular structure and intermolecular interactions on the hyperfine NMR shifts. However, to include supramolecular interactions in the theoretical calculations more rigorously and systematically, molecular dynamics simulations followed by quantum-chemistry calculations of the pNMR shifts should be performed.^41,42^ Research in this direction is underway in our laboratory.

## 3 Conclusions

We synthesized a set of symmetric Ru(III) and Rh(III) compounds containing 4-R-substituted pyridine ligands in the axial positions of the octahedral coordination sphere. The compounds were characterized using mass spectrometry, single-crystal X-ray diffraction, and NMR spectroscopy. To the best of our knowledge, ours is the first report of an X-ray structure of a symmetric Rh^III^Cl_4_L_2_ complex with *trans* arrangement of the axial pyridine-based ligands.

The effects of a paramagnetic Ru(III) center on the NMR shifts of ligand atoms were investigated using ^1^H and ^13^C NMR experiments at various temperatures. Complementary DFT calculations were used to assist with the assignment of the experimental NMR resonances and to investigate the nature of the NMR shifts. Specifically, we demonstrate the important roles played by (i) the 4-R substituent of the axial pyridine ligand, (ii) the structural *trans*-effect of the axial trans ligand, (iii) the coordination strength of the equatorial ligands, and (iv) the solvent on the distribution of spin density in the molecule as reflected in the NMR shifts of the atoms of pyridine ligands. Although we did not arrive at a full understanding of the solvent effects on the electronic structure and hyperfine NMR shifts, this represents an extremely interesting field for further investigation. The results we obtained systematically expand our previous pNMR studies of Ru(III) compounds,^8,9,30,31,40^ add new information about the distribution of spin density in open-shell molecules, and enhance our knowledge of hyperfine effects in transition-metal complexes in general.

## 4 Methods

### 4.1 Experimental Section

All of the reagents and starting compounds MCl_3_·*x*H_2_O (M = Ru, Rh), 4-methylpyridine (4-Me-Py), pyridine (Py), 4-phenylpyridine (4-Ph-Py), 4-trifluormethylpyridine (4-CF_3_-Py), 4-pyridinecarboxylic acid (4-COOH-Py), 3-pyridinecarboxylic acid (3-COOH-Py), 4-pyridinecarbonitrile (4-CN-Py), sodium/potassium tetraphenylborate (Na/KBPh_4_), tetraphenylphosphonium chloride (PPh_4_Cl), lithium chloride (LiCl), acetanhydride, acetic acid, silver sulfate, oxalic acid dihydrate (H_2_C_2_O_4_), and 1,2-^13^C2-oxalic acid (H_2_^13^C_2_O_4_) were used as obtained from commercial suppliers (ABCR, Alfa Aesar, Sigma-Aldrich, TCI Europe, Cambridge Isotope Laboratories). The solvents in p.a. grade were used with no further purification.

#### 4.1.1 Synthesis

**Figure 15:**
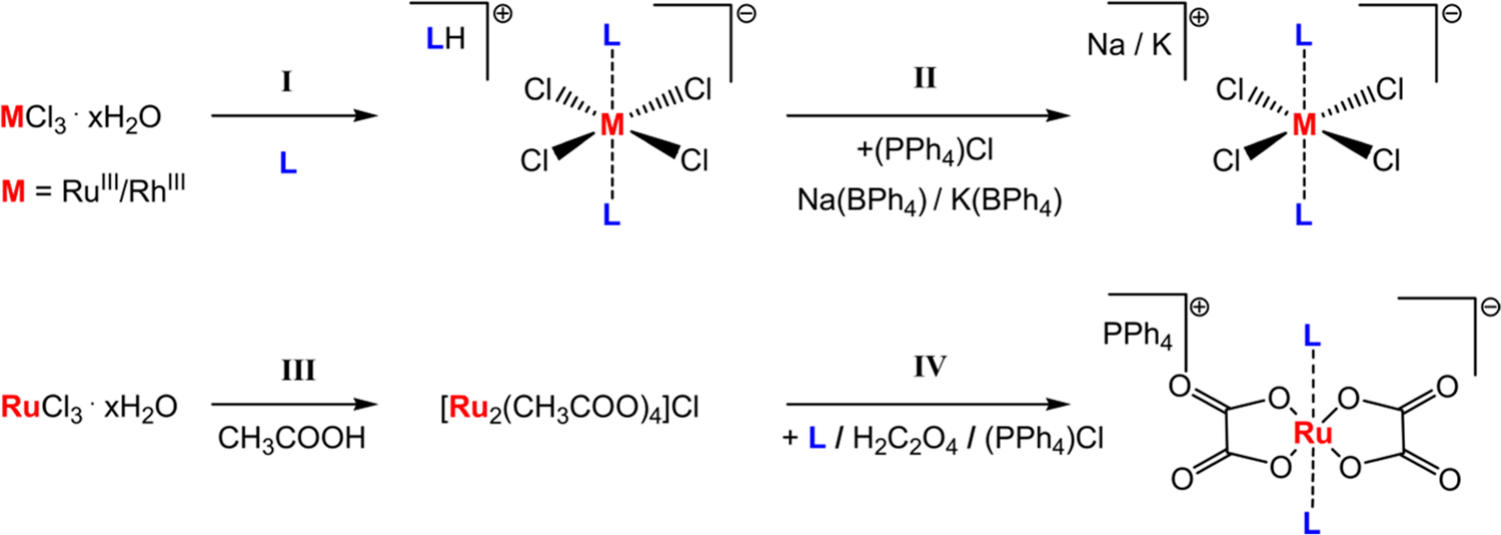
Schematic of the strategy used to synthesize symmetric Ru(III) and Rh(III) coordination compounds.

The synthetic pathway used to prepare coordination compounds with the general formula (Na^+^/K^+^/PPh_4_^+^/LH^+^) [*trans*-M^III^L(eq)*_n_*L(ax)_2_] (M = Ru(III) or Rh(III); L(eq) = Cl, *n* = 4; L(eq) = ox, *n* = 2; L(ax) = DMSO-*S*; 4-R-pyridine, R = Me, H, Ph, COOH, CF_3_, CN) is shown schematically in Figure 15.

The synthesis (**I**, **II**) of symmetric ruthenium and rhodium coordination compounds (**1**, **2**) corresponds to the preparation of KP-type Ru(III) compounds by previously reported synthetic procedures.^43,44^ The subsequent replacement of a protonated organic ligand by an alkali metal (Na^+^ or K^+^) cation was realized by direct reaction with NaBPh_4_ (KBPh_4_) reagent or after the prior transformation of coordination compounds into PPh_4_^+^ salts (**II**). Asymmetric ruthenium(III) and rhodium(III) compounds (**3**, **4**) were prepared using slightly modified forms of the synthetic procedures previously reported by Webb et al.^34^ and Mestroni et al.^45^ Ru(III) compounds **5** with two chelating oxalate ligands in the equatorial plane of the complex anion (including the ^13^C enriched sample) were prepared from a Ru_2_(μ-O_2_CCH_3_)_4_Cl precursor^46^ (**III**) by the modified synthetic procedure reported by Elnajjar et al.^47^ (**IV**). The coordination compounds were obtained as brown-orange (Ru^III^) or pink (Rh^III^) solids and characterized using NMR spectroscopy, mass spectrometry, and single-crystal X-ray diffraction.

#### 4.1.2 NMR Spectroscopy

The one-dimensional (1D) NMR spectra of Ru(III) and Rh(III) coordination compounds were measured on Bruker Avance Neo HD spectrometers (500 and 700 MHz). The NMR samples were prepared by dissolving 0.5–10 mg of the coordination compound in 0.5 mL of solvent (*N*,*N*-dimethyl-formamide-*d*_7_, dimethylsulfoxide-*d*_6_, or D_2_O). The NMR signals of the solvent (δ(^1^H) = 8.03 ppm for DMF-*d*_7_; δ(^1^H) = 2.50 ppm for DMSO-*d*_6_, and δ(^1^H) = 4.8 ppm for D_2_O) were used to reference the NMR spectra measured at various temperatures—the NMR shifts are reported relative to TMS.

#### 4.1.3 ESI-MS Spectrometry

Mass spectra were measured in the negative mode on a Q-TOF Impact II (Bruker Daltonics, Germany) mass spectrometer using electrospray ionization (ESI). Solid samples were dissolved in acetonitrile, acetone, or ultrapure water prior to analysis and subsequently diluted to a final concentration of 0.1 μg/μL. The samples were injected into the instrument at a flow rate of 300 μL/h. Parameters of the electrospray ion source were set as follows: end-plate offset 500 V with capillary voltage 4500 V, nebulizing nitrogen gas 0.4 bar, drying nitrogen gas 4 L/min, and drying-gas temperature 180 °C. MS spectra were acquired in the *m*/*z* range 100–800. For MS data, see Table S1 in the Supporting Information.

#### 4.1.4 X-ray Diffraction

Monocrystals for X-ray diffraction analyses were obtained via slow diffusion of diethyl ether vapors into acetonitrile solutions of Ru(III) and Rh(III) compounds **1f** and **2f** (both PPh_4_^+^ salts), by slow evaporation of the solvent from a solution of compound PPh_4_^+^**5a** in dichloromethane, or by cooling down the mother liquor of LH^+^**6d**. Diffraction data were collected on a Rigaku MicroMax-007 HF rotating anode four-circle diffractometer with Mo Kα radiation. The temperature during data collection was 120(2) K. The structures were solved by direct methods and refined by standard methods using the ShelXTL software package.^48^ Crystallographic data and structural refinement parameters are listed in Table 3.

**Table 3:** Selected Crystallographic Data for Compounds **1f**, **2f**, **5a** (all PPh_4_^+^ Salts), and LH^+^**6d**

	PPh_4_^+^**1f**	PPh_4_^+^**2f**	PPh_4_^+^**5a**	LH^+^**6d**
CCDC no.	2202835	2202834	2202833	2202832
chemical formula	C_38_H_31_Cl_4_N_5_PRu	C_38_H_31_Cl_4_N_5_PRh	C_41_H_36_Cl_2_N_2_O_8_PRu	C_18_H_16_Cl_4_N_3_O_6_Ru
formula weight	831.52	833.36	838.18	613.21
crystal system	monoclinic	monoclinic	triclinic	triclinic
space group	*P*2/*n*	*P*2/*n*	**P**1	*P*1
*a* (Å)	10.3713(7)	10.3329(7)	10.3503(3)	7.4257(10)
*b* (Å)	8.9224(6)	8.8658(6)	11.7867(5)	7.4958(9)
*c* (Å)	20.8055(15)	20.6917(14)	16.9671(5)	10.4153(13)
α (deg)	90	90	71.343(3)	76.99(2)
β (deg)	102.448(2)	102.877(2)	88.089(3)	75.88(3)
γ (deg)	90	90	82.139(3)	78.08(3)
*V* (Å^3^)	1880.0(2)	1847.9(2)	1942.60(12)	540.69(16)
*Z*	2	2	2	1
*D*_calcd._ (g cm^–3^)	1.469	1.498	1.518	1.883
μ (mm^–1^)	0.778	0.830	5.389	1.26
measured/unique reflections	15690/3319	10263/3042	24589/7159	5133/2431
data/parameters/restraints	3319/240/195	3042/240/182	7159/499/0	2431/291/240
*R*_1_/*w*R**_2_ [*I* > 2σ(*I*)]	0.0277/0.0755	0.0258/0.0720	0.0634/0.1715	0.0280/0.0756
*R*_1_/*w*R**_2_ [all data]	0.0300/0.0767	0.0267/0.0727	0.0638/0.1720	0.0299/0.0768
GoF	1.112	0.881	1.103	1.024
Δρ_max_/Δρ_min_ (e Å^–3^)	0.433/–0.438	0.396/–0.506	2.37/–1.61	0.55/–0.69

### 4.2 Theoretical

The geometries of the Ru(III) coordination compounds were optimized using a previously calibrated PBE0^49^/def2-TZVPP^50^/ECP/COSMO^51^ approach^8,52−56^ in the Turbomole 7.03 program.^38^ The effects of relativity on electronic structure and EPR parameters were included by zeroth-order regular approximation, ZORA.^57^ The electronic g-tensors and hyperfine coupling A-tensors^15,58^ were calculated at the unrestricted SO-ZORA/PBE0/TZ2P/COSMO level in ADF2019.^59^ Subsequently, the hyperfine NMR shifts (including δ^HFi^ and δ^HFa^)^9^ were obtained from **g** and **A** as reported previously.^8,10,16^ The calculation and analysis of δ^HFi^ and δ^HFa^ contributions in terms of FC (Fermi-contact), SD (spin-dipole), and PSO (paramagnetic spin–orbit) mechanisms^9,60−62^ were performed in the program ReSpect 5.2.0 (mDKS/PBE0/dyall-vtz^63^/pcJ-2^64^/COSMO).^65^ Spin densities and corresponding atomic and fragment spin populations were calculated at the unrestricted ZORA/PBE0/TZ2P/COSMO level in ADF2019.^59^

## Data Availability

The computational results are available in the ioChem-BD repository^66^ and can be accessed via https://doi.org/10.19061/iochem-bd-6-171.

## Supporting Information Available

Additional experimental and calculated NMR data and figures, MS, and X-ray data (PDF)
