# Supplementary material for: Nature of NMR Shifts in Paramagnetic Octahedral Ru(III) Complexes with Axial Pyridine-Based Ligands
Source: Inorg Chem. 2023 Feb 10;62(8):3381–94. doi: 10.1021/acs.inorgchem.2c03282 (PMC10017024; doi:10.1021/acs.inorgchem.2c03282)
Supplement: Supplementary file 1 — ic2c03282_si_001.pdf [file ic2c03282_si_001.pdf]

Supporting Information for

Nature of NMR Shifts in Paramagnetic Octahedral Ru(III) Complexes with Axial Pyridine-Based Ligands

Jan Chyba,[†,‡] Anna Hruzíková[†,‡] Michal Knor,[†,‡] Petra Pikulová,[†,‡] Kateřina Marková,[†,‡] Jan Novotný, *[†,‡,$,⁋] Radek Marek*[†,‡,$]

[†] *CEITEC – Central European Institute of Technology, Masaryk University, Kamenice 5, CZ-62500 Brno, Czechia*

[‡] *Department of Chemistry, Faculty of Science, Masaryk University, Kamenice 5, CZ-62500 Brno, Czechia*

[$] *National Center for Biomolecular Research, Faculty of Science, Masaryk University, Kamenice 5, CZ-62500 Brno, Czechia*

[⁋] *Institute of Inorganic Chemistry, Slovak Academy of Science, Dúbravská cesta 9, SK-84536 Bratislava, Slovakia*

* Email: jan.novotny@ceitec.muni.cz (J.N.), radek.marek@ceitec.muni.cz (R.M.)

Table S1. Calculated and observed masses (m/z) of the molecular signals in the ESI-MS(-) spectra of Ru(III) and Rh(III) coordination compounds **1a-6e**.

	Ru(III) compound			Rh(III) compounds	
	**Calc.**	**Observed**		**Calc.**	**Observed**
**1a**	429.8935	429.8936	**2a**	430.8937	430.8835
**1b**	401.8621	401.8615			
**1c**	553.9250	553.9239			
**1d**	489.8419	489.8395	**2d**	490.8421	490.8418
**1e**	537.8369	537.8369			
**1f**	451.8526	451.8506	**2f**	452.8529	452.8500
**5a**	463.9804	463.9799			
**5b**	435.9485	435.9484			
**5c**	588.0120	588.0114			
**5d**	523.9282	523.9257			
**6e**	489.8419	489.8414			

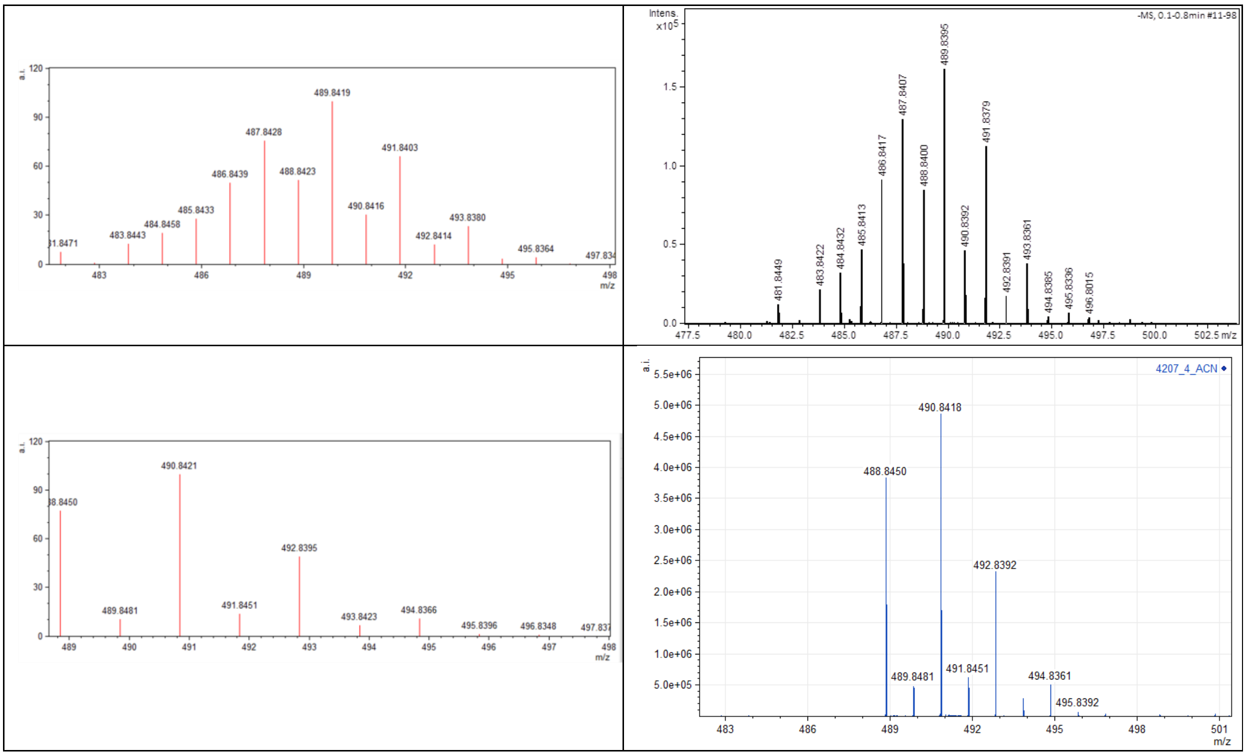

Figure S1. Calculated (left) and observed (right) isotopic patterns of the molecular signals in the ESI-MS(-) spectra of Ru(III) and Rh(III) compounds Na^+**1d** (top) and Na^+**2d** (bottom).

a)

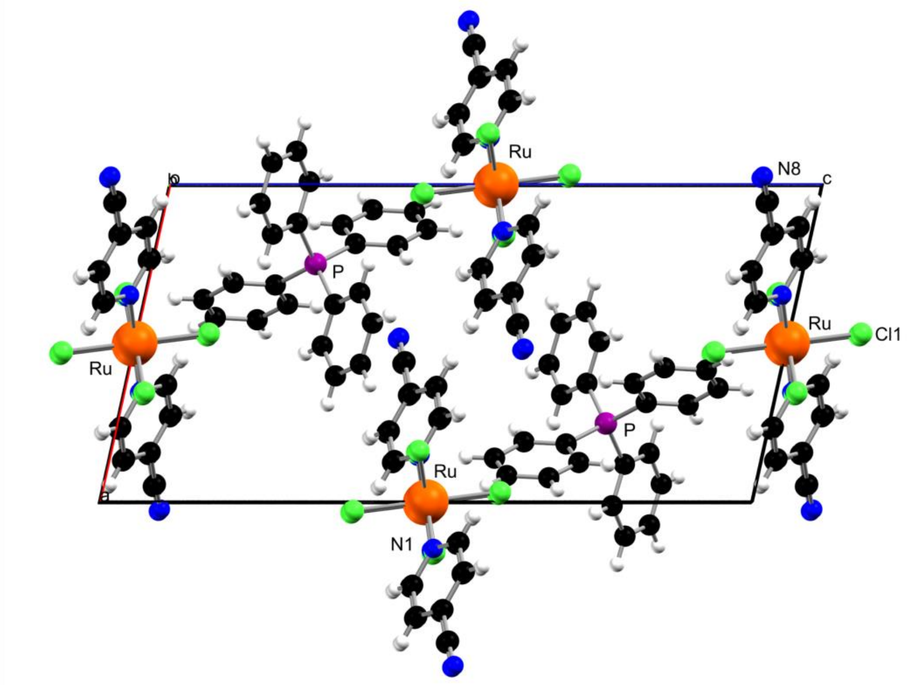

b)

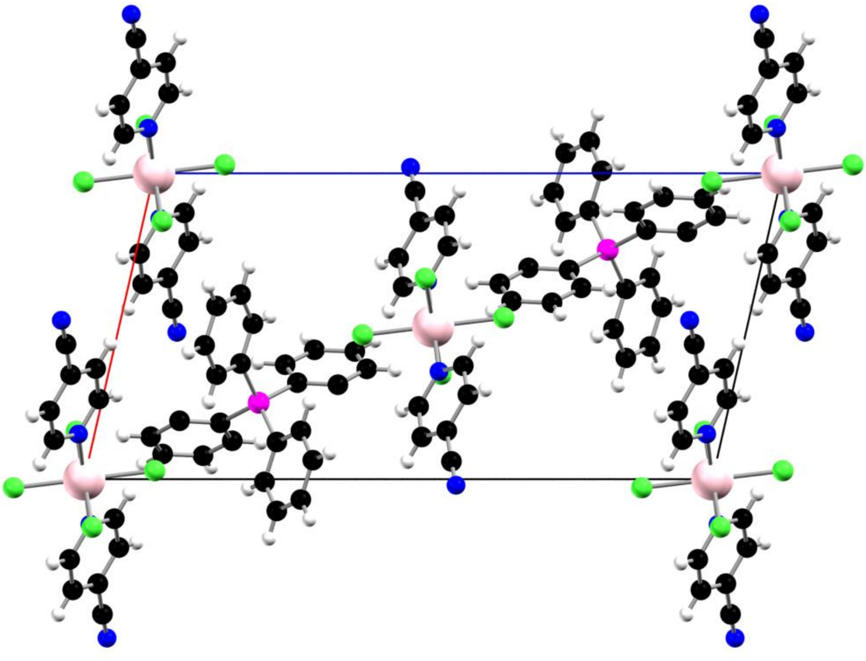

c)

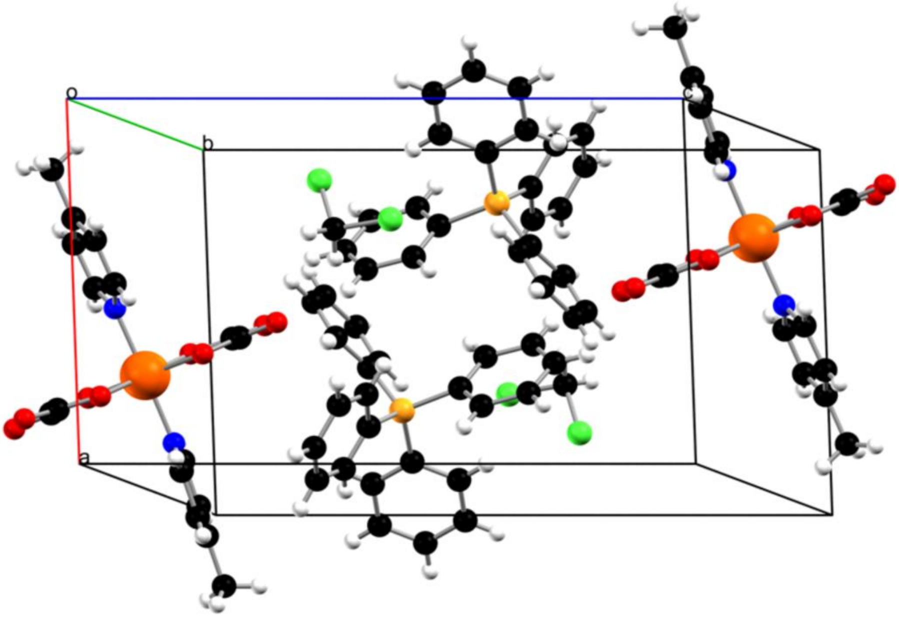

d)

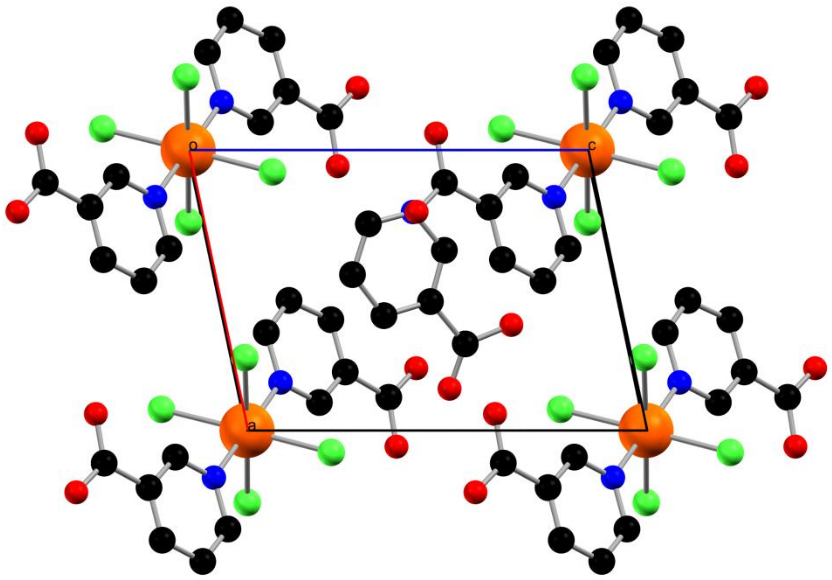

Figure S2. Crystal packing within the unit cell for compound a) PPh_4^+**1f** (CCDC No. 2202835), b) PPh_4^+**2f** (CCDC No. 2202834), c) PPh_4^+**5a** (CCDC No. 2202833), and d) LH^+**6d** (CCDC No. 2202832) determined by X-ray diffraction.

Table S2. Experimental ^{1}H NMR shifts ($\delta_{\mathrm{L}}^{\mathrm{tot}}$) for Ru(III) and Rh(III) complex anions **1a-6d** measured in DMSO-d_6 at 298 K.

Atom	**1a**	**1b**	**1c**	**1d**	**1e**	**1f**	**2a**	**2d**	**2f**
R	Me	H	C_6H_5	COOH	CF_3	CN	Me	COOH	CN
H2	-18.0	-17.0	-16.5	-15.5	-12.6	-12.4	+9.1	+9.2	+9.5
H3	-6.4	-6.3	-5.6	-5.9	-4.9	-5.2	+7.3	+7.8	+7.6
H(R)	-6.1	+3.6	+4.5, +5.5, +6.6	+11.1	-	-	+2.4	+13.9	-

Atom	**3d**	**4d**	**5a**	**5b**	**5c**	**5d**	**6d**
R	COOH	COOH	Me	H	C_6H_5	COOH	3-COOH
H2	-5.9	+9.4	-35.3	-34.9	-34.5	-32.5	-14.4 (+H6’)[a]
H3	-1.2	+7.9	-5.0	-5.6	-5.0	-5.8	-
H4’	-	-	-	-	-	-	+5.3
H5’	-	-	-	-	-	-	-5.9
H(R)	+12.3	+13.9	-2.6	+2.9	+3.8, +5.6, +6.3	+11.1	+13.6

[a] Signals of H2 and H6 unresolved.

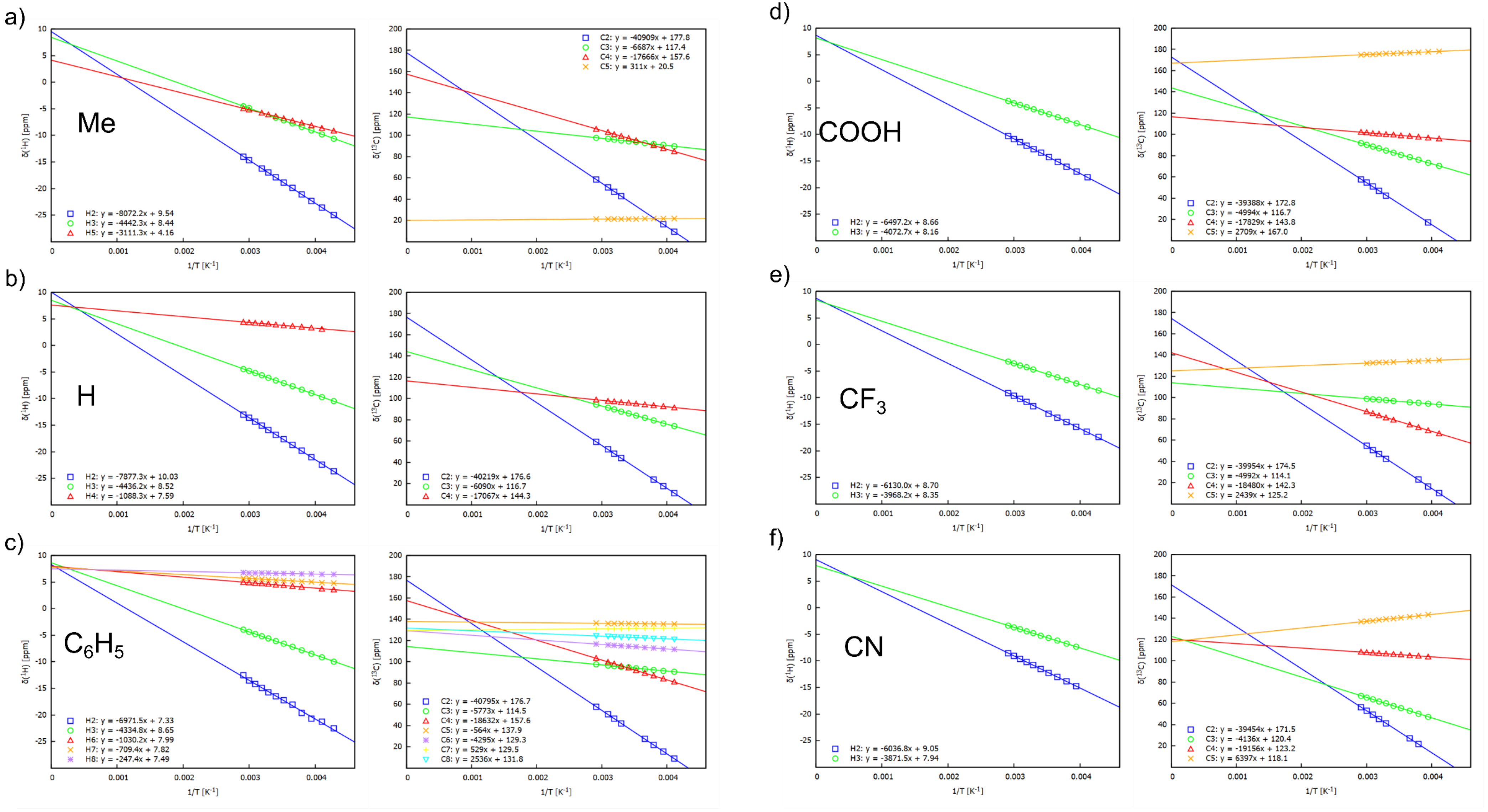

Figure S3. Curie plots for compounds a) **1a** (R = CH_3), b) **1b** (R = H), c) **1c** (R = Ph), d) **1d** (R = COOH), e) **1e** (R = CF_3), and f) **1f** (R = CN) measured in DMF-*d*$_7$.[1]

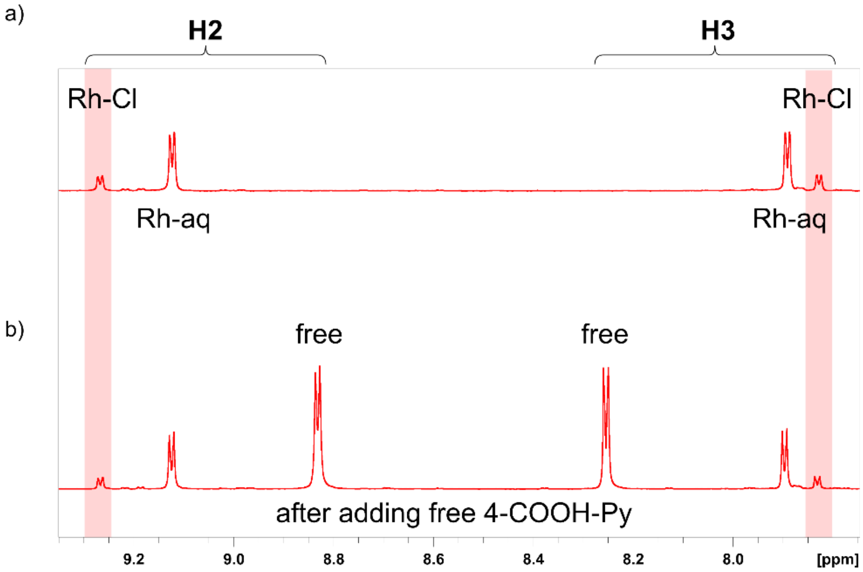

Figure S4. a) ^{1}H NMR spectrum of **2d** in D_2O (Note the dominating signals of an aqua form of the Rh(III) compound). b) ^{1}H NMR spectrum showing the position of free 4-COOH-pyridine after its addition to a solution of hydrolyzed **2d**.

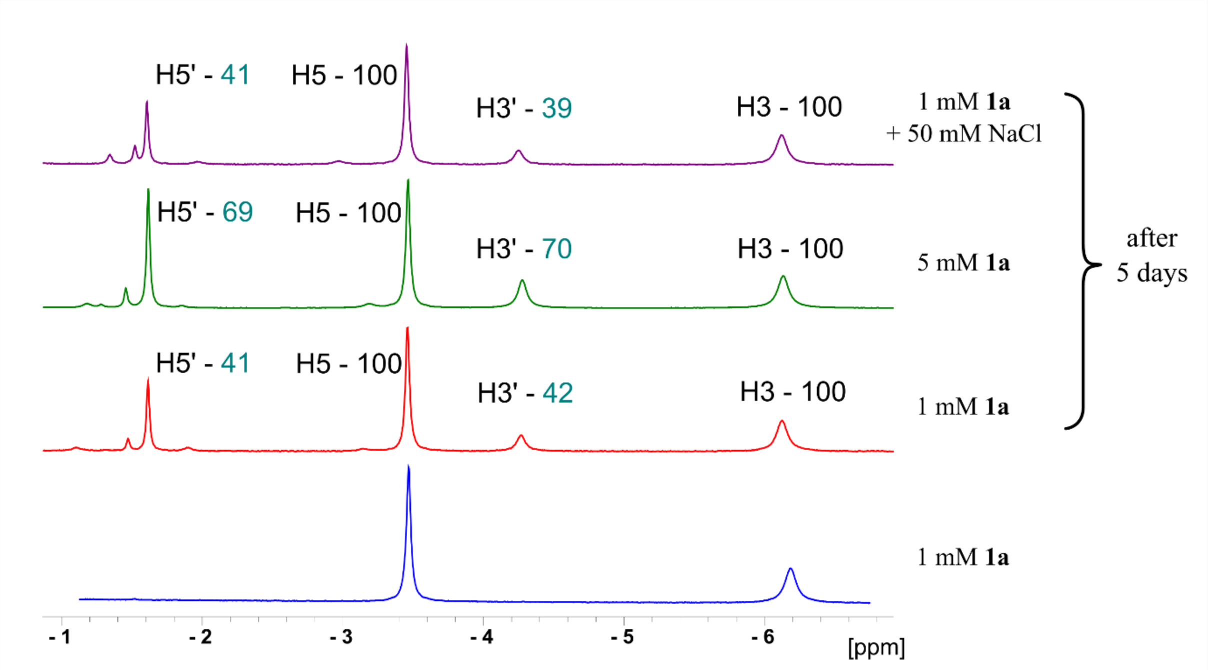

Figure S5. Detailed view of the paramagnetically shielded NMR resonances of K^+**1a** measured in aqueous solution at 298 K after 5 days at room temperature. The specific properties of the individual samples are indicated on the right. The relative population of new species resulting from Cl^- substitution (signals labelled with an apostrophe) is referenced to the original signals (labelled with 100). The rate of transformation is not dramatically affected by the ionic strength. In contrast a higher concentration of compound **1a** accelerates the solvolysis by 75%, probably because of autocatalytic activity[2,3] of the Ru(III) complex.

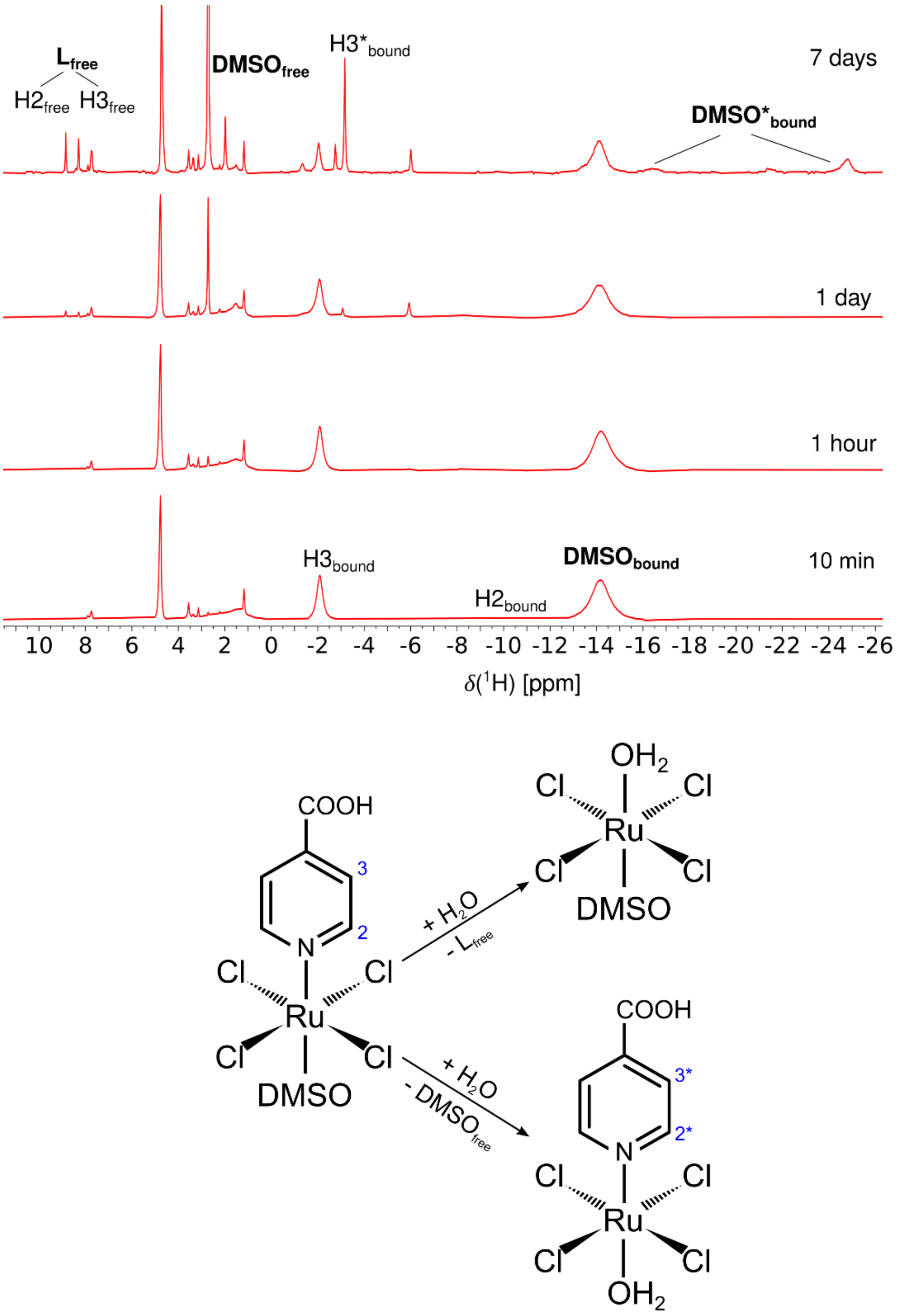

Figure S6. ^{1}H NMR spectra of compound **3d** measured at 298 K, 1 hour, 1 day, and 6 days after the sample preparation and the proposed hydrolysis of **3d** (decomposition path).

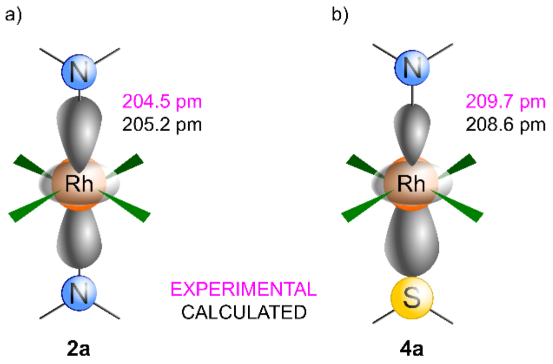

Figure S7. Schematic representation of the Rh–N bond in a) symmetric compound **2a** and b) asymmetric compound **4a** as optimized at the scalar-relativistic DFT level (PBE0/def2-TZVPP/COSMO-DMF) in the program Turbomole (black) and obtained from X-ray diffraction analysis (magenta).

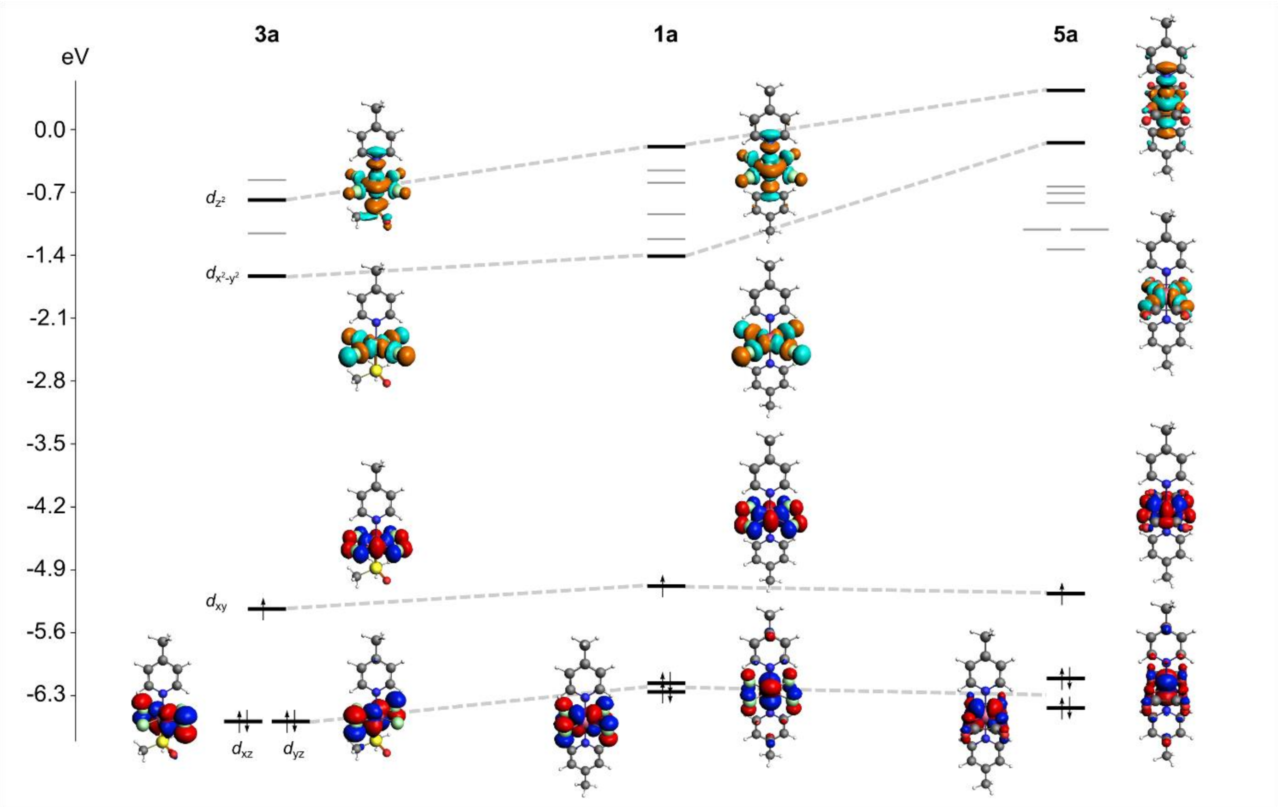

Figure S8. FMO diagram for compounds **3a**, **1a**, and **5a** calculated at the restricted PBE0/TZ2P/COSMO$_{water}$ level in the ADF program. Note that SOMO of metal d_{xy} character (delocalized to the equatorial ligands)[1] is similar for all three compounds. Therefore, there is only a slight change in the electronic g-tensor (see Table S7a).

Table S3. Experimental NMR shifts (δ) for compound **1a** and its aquated form, and DFT-calculated hyperfine shifts (δ^{HF}, SO-ZORA level, PBE0/TZ2P/COSMO-DMF/H_2O) for **1a**, **1a_aq**, and **1a-diaq** at 293 K. NMR shifts are given in ppm.

	Atom	δ (exp)	δ^{HF} (calc)			
		**1a (1a_aq?)**	**1a**	**1a**	**1a_aq**	**1a_diaq**
Charge		-1 (0)	(-1)	(-1)	(0)	(+1)
Solvent		H_2O	DMF	H_2O	H_2O	H_2O
^{1}H	H2	-23.8 (-20.7)	-19.9	-20.9	-22.1	-25.6
	H3	-6.2 (-4.2)	-15.2	-15.3	-13.7	-12.5
	H5	-3.5 (-1.6)	+2.2	+1.6	-0.8	-3.2
g-tensor	g_{11}	-	1.82	1.82	1.82	1.79
	g_{22}	-	**2.68**	**2.65**	**2.54**	**2.26**
	g_{33}	-	**2.71**	**2.71**	**2.79**	**3.19**

Table S4. Energy decomposition analysis of the Ru–N bond in compounds **1a** and **3a** (ZORA/PBE0/TZ2P/vac).

	**Ru-N**	
Energy term[a]	**1a**	**3a**
**TOT**	**-34.2**	**-32.6**
ELS	-102.4	-91.7
PAU	+116.9	+108.5
ORB	-48.6	-49.4

[a] Energy in kcal mol^{-1}.

Table S5. The DFT-calculated δ_{cal}^{HFi} and δ_{cal}^{HFa} contributions (SO-ZORA/PBE0/TZ2P/COSMO-DMF) to the hyperfine NMR shifts for compounds **1a**-**1f**. The NMR shifts are given in ppm at 293 K.

Atom	**1a** (Me)		**1b** (H)		**1c** (Ph)		**1d** (COOH)		**1e** (CF_3)		**1f** (CN)	
	δ^{HFi}	δ^{HFa}	δ^{HFi}	δ^{HFa}	δ^{HFi}	δ^{HFa}	δ^{HFi}	δ^{HFa}	δ^{HFi}	δ^{HFa}	δ^{HFi}	δ^{HFa}
H2	-4.4	**-16.8**	-4.3	**-15.9**	-3.9	**-16.2**	-1.8	**-13.0**	-2.2	**-13.3**	-1.2	**-12.5**
H3	-13.8	-0.6	-14.1	-1.0	-14.1	-0.8	-13.5	-1.1	-13.3	-1.0	-13.8	-1.3
H5	-	-	+4.1	-2.3	-	-	-	-	-	-	-	-
C2	**-130**	-10	**-132**	-10	**-136**	-11	**-139**	-11	**-137**	-11	**-141**	-11
C3	**-20**	-1	**-17**	-2	**-16**	-2	**-7**	0	**-10**	-1	**-4**	0
C4	**-51**	-9	**-51**	-9	**-57**	-9	**-64**	-10	**-64**	-10	**-72**	-10
C5	**+9**	-2	-	-	**+20**	-2	**+22**	-2	**+21**	-2	**+45**	-1
H6	-	-	-	-	0	-1.8	-	-	-	-	-	-
H7	-	-	-	-	-1.9	0	-	-	-	-	-	-
H8	-	-	-	-	+1	0	-	-	-	-	-	-
C6	-	-	-	-	-17	-2	-	-	-	-	-	-
C7	-	-	-	-	+6	-1	-	-	-	-	-	-
C8	-	-	-	-	-10	-2	-	-	-	-	-	-

Table S6. The experimental ($\delta^{\mathrm{HF}}_{\mathrm{exp}}$, in D_2O) and calculated ($\delta^{\mathrm{HF}}_{\mathrm{cal}}$, SO-ZORA/PBE0/TZ2P/COSMO-water) hyperfine contributions (temperature dependent) to the NMR shift (in ppm) for K^+[*trans*-$Ru^{III}Cl_4L_2]^-$ compound **1a** and its LH^+[*trans*-$Ru^{III}Cl_4$(DMSO-*S*)L$]^-$ analog **3a** at 298 K. The calculated values of the isotropic hyperfine shifts are decomposed into contributions from isotropic ($\delta^{\mathrm{HFi}}_{\mathrm{cal}}$) and anisotropic ($\delta^{\mathrm{HFa}}_{\mathrm{cal}}$) hyperfine interactions (cations were neglected in the calculations).

	K^+[*trans*-$Ru^{III}Cl_4L_2]^-$ (**1a**)			LH^+[*trans*-$Ru^{III}Cl_4$(DMSO-*S*)L$]^-$ (**3a**)[a]		
	$\boldsymbol{\delta^{\mathrm{HF}}_{\mathrm{exp}}}$	$\delta^{\mathrm{HF}}_{\mathrm{cal}}$	$\delta^{\mathrm{HFi}}_{\mathrm{cal}}/\delta^{\mathrm{HFa}}_{\mathrm{cal}}$	$\boldsymbol{\delta^{\mathrm{HF}}_{\mathrm{exp}}}$	$\delta^{\mathrm{HF}}_{\mathrm{cal}}$	$\delta^{\mathrm{HFi}}_{\mathrm{cal}}/\delta^{\mathrm{HFa}}_{\mathrm{cal}}$
H2	**-36.2**	-22.2	-4.7/-17.5	**-19.2**	-15.0	-7.1/-7.9
H3	**-18.1**	-14.5	-14.0/-0.5	**-11.3**	-9.6	-9.9/+0.3
H5	**-11.1**	-10.7	-9.5/-1.3	**-6.1**	-5.9	-5.0/-0.9
C2	-	-140	-130/-10	-	-74	-73/-1
C3	-	-24	-23/-2	-	-28	-26/-2
C4	-	-59	-50/-9	-	-28	-24/-4
C5	-	+6	+8/-2	-	-2	+3/-1

[a] Data from Ref. 1.

Table S7a. Decomposition of the isotropic values of the A-tensor (in MHz) calculated at 4c/PBE0/dyall-vtz/pcJ-2/COSMO-DMF level in the program ReSpect (ver. 5.2)[4] and the corresponding contributions to the hyperfine NMR shifts (in ppm) at 293 K.[a]

	**1a**			**3a**			**5a**		
	A_{iso}^{FC}	A_{iso}^{PSO}	A_{iso}^{SD}	A_{iso}^{FC}	A_{iso}^{PSO}	A_{iso}^{SD}	A_{iso}^{FC}	A_{iso}^{PSO}	A_{iso}^{SD}
H2	**+0.08**	**-0.25**	-0.06	**-0.08**	**-0.15**	-0.02	**+0.44**	**-0.39**	**-0.12**
H3	**-0.26**	-0.11	-0.02	**-0.20**	-0.06	-0.01	**-0.26**	-0.14	-0.03
H5	**-0.28**	-0.06	-0.01	**-0.15**	-0.03	-0.00	**-0.21**	-0.05	-0.01
C2	**-0.88**	+0.03	-0.16	**-0.48**	+0.00	-0.05	**-1.18**	-0.18	-0.16
C3	**-0.04**	-0.06	+0.02	**-0.13**	-0.03	+0.00	**+0.21**	-0.08	+0.01
C4	**-0.36**	-0.08	-0.05	**-0.18**	-0.03	-0.02	**-0.34**	-0.01	-0.09
C5	**+0.11**	0.00	-0.02	**+0.05**	-0.01	-0.00	**+0.15**	-0.01	-0.01
	$\delta_{iso/ani}^{FC}$	$\delta_{iso/ani}^{PSO}$	$\delta_{iso/ani}^{SD}$	$\delta_{iso/ani}^{FC}$	$\delta_{iso/ani}^{PSO}$	$\delta_{iso/ani}^{SD}$	$\delta_{iso/ani}^{FC}$	$\delta_{iso/ani}^{PSO}$	$\delta_{iso/ani}^{SD}$
H2	+2.31/ -0.32	-7.31 / -0.82	-1.82 / -9.09	-2.42 / -0.05	-4.43 / -0.29	-0.57 / -4.81	+11.9/-0.17	-10.81/-3.09	-3.41/-14.03
H3	-7.59 / +0.13	-3.21 / -0.19	-0.70 / -2.79	-5.87 / +0.02	-1.81 /-0.09	-0.21 / -1.70	--7.09/+0.09	-3.81/-0.39	-0.87/-3.38
H5	-7.97 / +0.23	-1.71 / -0.10	-0.41 / -1.50	-4.32 / +0.04	-0.98 / -0.05	-0.13 / -0.91	-7.56/-0.01	-1.80/-0.14	-0.47/-1.64
C2	-100.8 / +2.4	+3.5 / -3.1	-18.4 / -21.0	-55.5 / +0.3	+0.2 / -0.4	-5.4 / -9.5	-128.5/+8.6	-19.6/-6.6	-17.8/-30.8
C3	-4.4 / -1.0	-6.8 / -0.5	+1.8 / -0.7	-15.7 / -0.2	-3.8 /-0.2	+0.4 /-1.7	+22.8/+0.9	-9.1/-0.9	+1.2/-0.2
C4	-41.9/+1.1	-6.1/+0.3	-8.8/-11.9	-21.3 / +0.2	-3.6 / 0.0	-2.3 / -4.3	-37.6/-0.2	-1.4/-0.2	-9.4/-16.5
C5	+13.1/-0.4	-2.0/-0.2	-0.8/-2.0	+5.6 / -0.1	-1.3 / -0.1	-0.3 / -1.2	+16.1/0.0	-1.2/-0.2	-0.9/-2.2

[a] Electronic **g**-tensor values are 1.51, 2.44, 2.50 for **1a**, 1.85, 2.34, 2.37 for **3a**, and 1.34, 2.10, 2.69 for **5a**.

Table S7b. Contributions to the hyperfine NMR shifts (in ppm) for compounds **1a** and **1f** calculated at the 4c/PBE0/dyall-vtz/pcJ-2/COSMO-DMF level in the program ReSpect (ver. 5.2)[4] at 293 K.

	**1a**				**1f**			
	$\delta_{iso/ani}^{FC}$	$\delta_{iso/ani}^{PSO}$	$\delta_{iso/ani}^{SD}$	$\delta_{iso/ani}^{TOT}$	$\delta_{iso/ani}^{FC}$	$\delta_{iso/ani}^{PSO}$	$\delta_{iso/ani}^{SD}$	$\delta_{iso/ani}^{TOT}$
H2	+2.31/ -0.32	-7.31 / -0.82	-1.82 / -9.09	**-6.82/-10.2**	+5.30/-0.13	-6.36/-0.86	-1.51/-7.63	**-2.57/-8.62**
H3	-7.59 / +0.13	-3.21 / -0.19	-0.70 / -2.79	**-11.48/-2.84**	-8.83/+0.07	-2.55/-0.18	-0.56/-2.20	**-11.95/-2.32**
C2	-100.8 / +2.4	+3.5 / -3.1	-18.4 / -21.0	**-115.2/-21.7**	-115.3/+1.3	+3.8/-1.9	-13.5/-18.4	**-124.5/-19.0**
C3	-4.4 / -1.0	-6.8 / -0.5	+1.8 / -0.7	**-9.3/-2.2**	+8.5/-0.3	-6.3/-0.3	+1.9/+0.4	**+4.2/-0.2**
C4	-41.9/+1.1	-6.1/+0.3	-8.8/-11.9	**-56.6/-10.5**	-62.8/+0.3	-5.0/+0.1	-6.3/-11.8	**-73.8/-11.4**
C5	+13.1/-0.4	-2.0/-0.2	-0.8/-2.0	**+10.2/-2.6**	+47.7/-0.2	-1.7/-0.1	0.0/+0.3	**+45.8/0.0**

Table S8. Experimental ^{1}H NMR shifts (in ppm) for compounds Na^+**1a** and Na^+**5a** in DMF-d_7, D_2O, and their mixtures (volume fraction of D_2O in DMF-d_7, ϕ_{vol} = 0, 0.25, 0.50, 0.75, and 1.0).

ϕ_{vol} (D_2O)	Na^+1a			Na^+5a		
	H2	H3	Me	H2	H3	Me
0 (DMF-d_7)	-17.13	-6.36	-6.27	-34.61	-5.37	-3.16
0.25	-19.42	-6.73	-5.97	-37.94	-3.82	-0.61
0.50	-21.59	-6.90	-5.43	-38.69	-1.96	1.97
0.75	-23.17	-6.84	-4.79	-38.23	-0.04	4.49
1.00 (D_2O)	-24.32	-6.58	-3.91	-36.74	2.61	8.02

Table S9. Changes in the experimental ^{1}H NMR shifts (Δδ in ppm) for compounds Na^+**1a** and Na^+**5a** induced by modification of the solvent composition (D_2O in DMF-d_7, ϕ_{vol} = 0, 0.25, 0.50, 0.75, and 1.0).

ϕ_{vol} (D_2O)	Na^+1a			Na^+5a		
	H2	H3	Me	H2	H3	Me
0	0	0	0	0	0	0
0.25	-2.29	-0.37	0.30	-3.33	1.55	2.55
0.50	-4.46	-0.54	0.84	-4.08	3.41	5.13
0.75	-6.04	-0.48	1.48	-3.62	5.33	7.65
1.00	-7.19	-0.22	2.36	-2.13	7.98	11.18

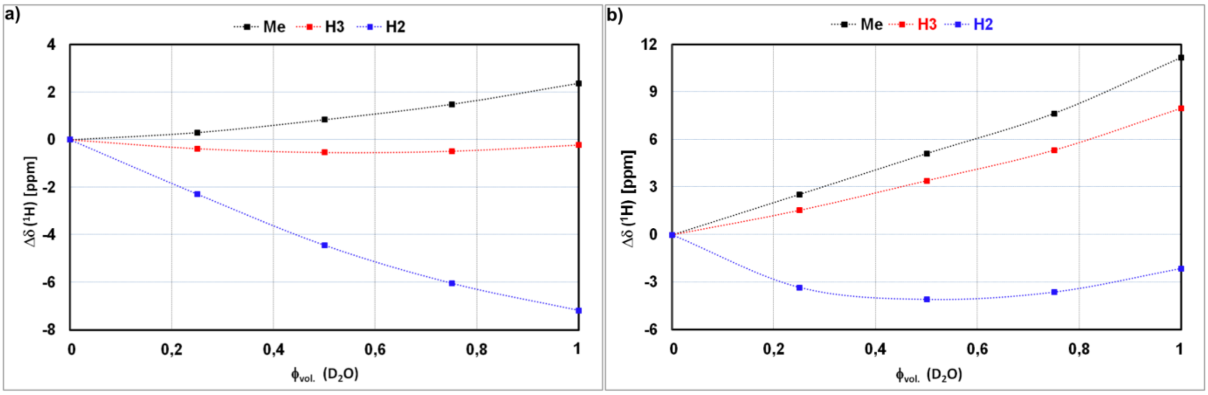

Figure S9. Effect of the solvent composition (D_2O in DMF-d_7, ϕ_{vol} = 0, 0.25, 0.50, 0.75, and 1.0) on the ^{1}H NMR shifts for a) compound Na^+**1a** and b) compound Na^+**5a**.

REFERENCES

(1) Novotný, J.; Sojka, M.; Komorovsky, S.; Nečas, M.; Marek, R. Interpreting the Paramagnetic NMR Spectra of Potential Ru(III) Metallodrugs: Synergy between Experiment and Relativistic DFT Calculations. *J. Am. Chem. Soc.* **2016**, *138* (27), 8432–8445. https://doi.org/10.1021/jacs.6b02749.

(2) Webb, M. I.; Walsby, C. J. Control of Ligand-Exchange Processes and the Oxidation State of the Antimetastatic Ru(Iii) Complex NAMI-A by Interactions with Human Serum Albumin. *Dalton Trans.* **2011**, *40* (6), 1322. https://doi.org/10.1039/c0dt01168a.

(3) Bacac, M.; Hotze, A. C. G.; Schilden, K. van der; Haasnoot, J. G.; Pacor, S.; Alessio, E.; Sava, G.; Reedijk, J. The Hydrolysis of the Anti-Cancer Ruthenium Complex NAMI-A Affects Its DNA Binding and Antimetastatic Activity: An NMR Evaluation. *Journal of Inorganic Biochemistry* **2004**, *98* (2), 402–412. https://doi.org/10.1016/j.jinorgbio.2003.12.003.

(4) Repisky, M.; Komorovsky, S.; Malkin, V.; Malkina, O. L.; Kaupp, M.; Ruud, K.; Bast, R.; Ekstrom, U.; Kadek, M.; Knecht, S.; Konecny, L.; Malkin, E.; Malkin Ondík, I. *Relativistic Spectroscopy DFT Program ReSpect, Developer Version 5.2.0*; www.respectprogram.org, 2020.
